# Road to Extinction? Past and Present Population Structure and Genomic Diversity in the Koala

**DOI:** 10.1093/molbev/msaf057

**Published:** 2025-03-25

**Authors:** Binia De Cahsan, Marcela Sandoval Velasco, Michael V Westbury, David A Duchêne, Mikkel H Strander Sinding, Hernán E Morales, Daniela C Kalthoff, Ian Barnes, Selina Brace, Roberto Portela Miguez, Alfred L Roca, Alex D Greenwood, Rebecca N Johnson, Matthew J Lott, M Thomas P Gilbert

**Affiliations:** Globe Institute, University of Copenhagen, 1350 Copenhagen K, Denmark; Globe Institute, University of Copenhagen, 1350 Copenhagen K, Denmark; Center for Genome Sciences (CCG), National Autonomous University of Mexico (UNAM), Cuernavaca, Mexico; Globe Institute, University of Copenhagen, 1350 Copenhagen K, Denmark; Globe Institute, University of Copenhagen, 1350 Copenhagen K, Denmark; Department of Biology, University of Copenhagen, DK-2200 Copenhagen N, Denmark; Globe Institute, University of Copenhagen, 1350 Copenhagen K, Denmark; Department of Zoology, Swedish Museum of Natural History, SE-104 05 Stockholm, Sweden; Department of Earth Sciences, Natural History Museum, London SW7 5BD, England, UK; Department of Earth Sciences, Natural History Museum, London SW7 5BD, England, UK; Department of Life Sciences, Natural History Museum, London SW7 5BD, England, UK; Department of Animal Sciences, University of Illinois, Urbana, IL 61801, USA; Department of Wildlife Diseases, Leibniz Institute for Zoo and Wildlife Research, 10315 Berlin, Germany; Department of Veterinary Medicine, Freie Universität Berlin, 14163 Berlin, Germany; Smithsonian National Museum of Natural History, Washington, D.C. 20560, USA; Australian Centre for Wildlife Genomics, Australian Museum, Sydney, NSW 2010, Australia; Globe Institute, University of Copenhagen, 1350 Copenhagen K, Denmark; Norwegian University of Science and Technology, University Museum, 7491 Trondheim, Norway

**Keywords:** Koalas, population genomics, conservation genomics, ancient DNA

## Abstract

Koalas are arboreal herbivorous marsupials, endemic to Australia. During the late 1800s and early 1900s, the number of koalas declined dramatically due to hunting for their furs. In addition, anthropogenic activities have further decimated their available habitat, and decreased population numbers. Here, we utilize 37 historic and 25 modern genomes sampled from across their historic and present geographic range, to gain insights into how their population structure and genetic diversity have changed across time; assess the genetic consequences of the period of intense hunting, and the current genetic status of this iconic Australian species. Our analyses reveal how genome-wide heterozygosity has decreased through time and unveil previously uncharacterized mitochondrial haplotypes and nuclear genotypes in the historic dataset, which are absent from today's koala populations.

## Introduction

The current biodiversity crisis is marked by a concerning increase in species loss, primarily attributed to anthropogenic activities like habitat destruction, climate change, impacts of urbanization, and overhunting (Pievani 2014; Freedman 2018). These activities are driving many species to the brink of extinction, with consequences that extend beyond individual losses, affecting entire ecosystems and threatening the intricate web of life. The impact of these declines extends to genetic processes, placing populations at risk of substantial genetic drift, inbreeding depression, and reduced adaptability (Caughley 1994; Reed and Frankham 2003; Frankham 2005; Ceballos et al. 2017). Consequently, this raises concerns about the long-term resilience of these populations to environmental changes (Caughley 1994).

In the last decade, the use of high-throughput sequencing techniques has enabled the profiling of current genome-wide diversity at the population level, providing valuable insights into present-day genetic variation, inbreeding levels, and the presence of deleterious alleles (Garner et al. 2016; Eusebi et al. 2019). However, a complementary method that is gaining increased interest as a tool for assessing the genetic challenges endangered species are facing, involves examining genomic data sampled over time, moving away from sole reliance on present-day genetic information (Díez-Del-Molino et al. 2017; Jensen et al. 2022). By directly comparing contemporary genomic data with that derived from historic specimens collected before current demographic declines, it becomes possible to quantify recent alterations in genetic parameters across the entire genome, in particular genomic erosion (Díez-Del-Molino et al. 2017) (i.e. the gradual loss of genetic diversity within a population or a species due to a reduction in the variety of genes and alleles present in the gene pool, due to increased inbreeding, small population sizes, and genetic drift). Such information can then contribute toward more comprehensive assessments of threat levels in endangered species (Der Sarkissian et al. 2015; Palkopoulou et al. 2015; Yeates et al. 2016). Therefore, historical specimens can serve as invaluable resources, offering baseline levels of diversity, inbreeding, and genetic load, which is why museums and other repositories of biological material are becoming an increasingly significant component of conservation efforts for threatened species (Poo et al. 2022). These collections provide crucial data to understand past genetic diversity and help inform future conservation strategies.

**Fig. 1.**
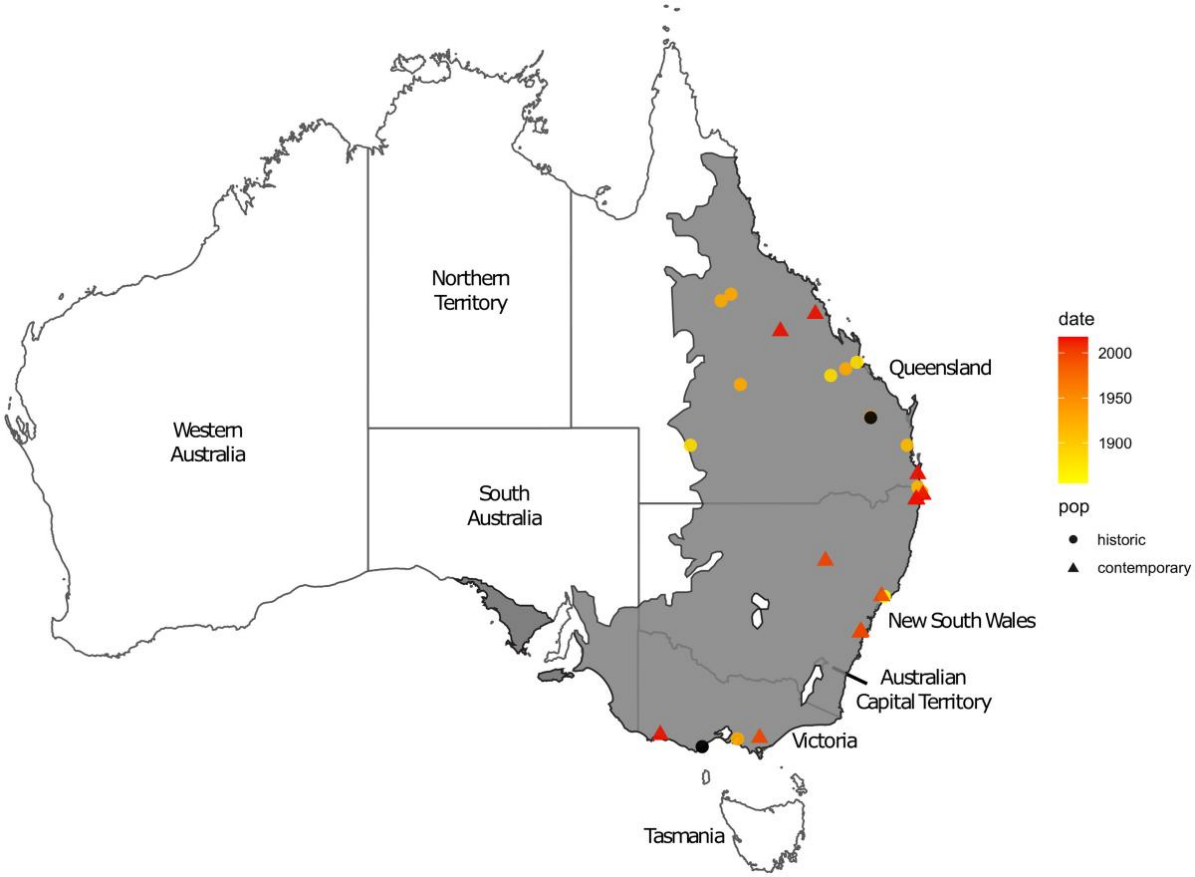
*Map of the Australian continent showing sample locations*. The maximum estimated contemporary distribution of koalas across states of QLD, NSW, (including the ACT), VIC, and SA (Adams-Hosking et al. 2016), with the origin of specimens analyzed in this study (colored triangles for contemporary samples and dots for historical samples). The color gradient from deep red (youngest) to light yellow (oldest) is indicative of the specimen's age. Black circles highlight samples of unknown age.

The koala (*Phascolarctos cinereus*) is a distinctive arboreal herbivorous marsupial endemic to the temperate, subtropical and tropical forests, and moist to semiarid woodlands of eastern and southern Australia. Despite their cultural significance and enduring popularity, koalas have been heavily impacted by anthropogenic activities and their downstream effects over the past few centuries, including hunting, habitat loss, climate change, altered fire regimes, modified host-pathogen dynamics, predation by exotic carnivores, competition with introduced herbivores, and vehicle collisions (McAlpine et al. 2015; Adams-Hosking et al. 2016; Park and Roberts 2022 [DAWE]). By 2012, the subsequent population declines had led to koalas being classified as “Vulnerable” under the Heffernan et al (1999) (EPBC Act) in the states of Queensland (QLD), New South Wales (NSW), and the Australian Capital Territory (ACT) (Australian Senate, Environment and Communications References Committee 2011; Shumway et al. 2015). Less than a decade later, in 2021, the status of these same populations was changed to “Endangered” (Park and Roberts 2021 [TSSC]). Conversely, koala populations in the states of Victoria (VIC) and South Australia (SA) are excluded from this listing, being widely considered stable, or even overabundant in some cases. This situation highlights the complex management challenge presented by koalas (Lott et al. 2024). Among these challenges is the extreme difficulty associated with directly monitoring regional shifts in the abundance and genetic health of threatened populations. At present, koalas inhabit a broad area of eastern mainland Australia, ranging from north-east QLD to the south-eastern tip of SA (Fig. 1). However, abundance data for many regions remain patchy and incomplete. Furthermore, the large temporal and geographical gaps in existing records make it difficult to confidently assess the impacts of current or emerging threats on specific populations across the koala's broad distribution (Adams-Hosking et al. 2016). Collecting this information is critical for the development of evidence-based management paradigms that make efficient use of relatively limited resources to stabilize or rehabilitate declining koala populations. Fortunately, genomic tools provide an avenue to not only directly assess genetic responses to key threatening processes or targeted conservation actions through the comparison of historical and contemporary specimens, but also to reconstruct both recent and ancient demographic trends for populations where the fragmentation or extirpation of local populations has rendered traditional survey methods impractical or impossible. This is particularly important for koalas at the extreme western edge of the species’ distribution, where population declines have been the most severe and contemporary sampling is proportionately difficult (Adams-Hosking et al. 2016). From approximately the 1890s, there was a surge in hunting for their pelts, due to a tremendous increase in demand for the domestic and international fur trade (Martin and Handasyde 1999; Anon 2014). For example, it has been estimated that during the hunting peak in 1924, over two million individuals were killed in QLD alone (Martin and Handasyde 1999). This led to drastic population declines, and ultimately resulted in the extirpation of many populations by the 1930s, most notably in SA (Phillips 1990; Martin and Handasyde 1999). Coupled with this was the historic challenge of extensive reduction in their natural habitat (Gordon et al. 2006), which continues to the present day as habitat is cleared for housing and other urban developments. In addition, koalas have been threatened by two well documented and widespread pathogens, the *Chlamydiaceae* bacteria and the koala retrovirus (KoRV). Both pathogens are linked to debilitating diseases, including conjunctivitis, blindness, infertility, and increased cancer risk. A potentially contributing factor to the severity of these diseases is the limited genetic diversity that may have resulted from historical population bottlenecks and habitat loss, as has been reported in other species (Pearman and Garner 2005; Whiteman et al. 2006).

It has been shown that chlamydial disease progression in koalas is linked to specific immune gene variants (Robbins et al. 2020), which can become fixed through inbreeding in small populations. Similarly, high levels of oxalate nephrosis in southern koalas are attributed to the fixation of deleterious alleles in bottlenecked populations. Unlike their northern counterparts, southern koalas exhibit low levels of full-length KoRV and lower Chlamydia prevalence, underscoring regional differences in disease susceptibility and genetic health (Speight et al. 2013).

Various molecular markers have previously been employed to assess the genetic variability of contemporary wild koala populations. Early studies based on mitochondrial DNA (mtDNA)-RFLPs and microsatellite data indicated low genetic diversity within, and limited gene flow between, koala populations (Taylor et al. 1991, 1997; Timms et al. 1993; Houlden et al. 1996). Notably, these studies also revealed substantial genetic differentiation among koala populations in QLD and NSW, contrasting with minimal differentiation in populations from VIC. This has been attributed to the recent ancestry of VIC's koalas from a small number of individuals originating from the French and Philip Islands (Houlden et al. 1999). A more recent mtDNA study of 662 koalas sampled throughout their distribution, found that koala control region (CR) haplotypes were divided into four weakly differentiated lineages, which correspond to three geographic clusters separated by Pleistocene biogeographic barriers: a central lineage, a southern lineage, and two northern lineages co-occurring north of Brisbane (Neaves et al. 2016). However, genetic data from historic specimens offer a unique perspective into the factors that have shaped the genetic diversity of koalas throughout their evolutionary history. For example, a study from 2012 investigated part of the hypervariable region of mtDNA in koala museum specimens collected in the 19th and 20th centuries and found that the mtDNA haplotypes observed in historical museum samples mirrored those found in contemporary koala populations, with no identification of novel haplotypes. Thus, the authors concluded that low mtDNA diversity may have been present in koala populations prior to recent population declines (Tsangaras et al. 2012).

Given that mtDNA only traces the maternal lineage and serves as a single genetic marker, it may not fully represent the complex genetic history of a species, necessitating the use of additional markers (Anon 1989). In this regard, a more recent genetic study utilized genome-wide SNP markers profiled in 171 koalas from eight populations across their range, to assess their genomic variability and population structure (Kjeldsen et al. 2016). These results identified significant broad-scale genetic differentiation between all the geographically separated populations investigated, with the greatest divergence observed between QLD, NSW, and VIC. As such, the species’ genetic diversity might not be as low as previously thought when compared to other vertebrates (Houlden et al. 1996; Tsangaras et al. 2012; Kjeldsen et al. 2016). More recently Lott et al. (2022) investigated ∼250 contemporary koala exomes from 91 locations across the species range. These authors found that current genome-wide diversity reveals no notable differentiation between genetic clusters of koalas, but identified a distinct geographical trend across the continent, showing a gradual decrease in genetic diversity in southern populations. Furthermore, the study revealed that koalas in the southern regions (VIC and SA) show lower genetic diversity, likely due to past events like excessive hunting, relocations from inbred populations, and overall population declines. On the other hand, according to this study, koalas in the northern areas (QLD and NSW) appear to have evaded extreme population reductions despite past hunting pressures, as indicated by their examination of museum samples collected over the past 118 year. The study also identified five major genetic clusters across the continent, historically separated by several biogeographic barriers (Lott et al. 2022), and provided data on koalas from populations, such as those from the Northern Beaches of Sydney, which are now believed to have been extirpated.

Previous studies have laid a foundational and informative groundwork using methods like ddRAD or exome capture. However, whole-genome sequencing (WGS) offers a more comprehensive approach by examining a larger proportion of the genome, including important regulatory, neutral, and noncoding regions (Hohenlohe et al. 2021). WGS allows for a broader and more detailed view of genetic diversity, capturing rare variants specific to certain populations that may be missed by methods focusing only on exons and enzyme restriction sites. This comprehensive analysis is crucial for understanding the full spectrum of genetic variation, evolutionary history, and population dynamics. By incorporating both historic and contemporary genomes, WGS can enhance our insights into adaptive traits and the subtleties of genetic drift, therefore providing a deeper and more nuanced understanding of a species’ genetic makeup.

To address this need, we elected to explore the genomic diversity and population structure of koalas across different time periods, with a focus on historic specimens predating the koala's recent population decline. By comparing historical genomic data with contemporary samples, this research aims to elucidate how past events, such as hunting and habitat loss, have influenced the genetic makeup of koala populations. Furthermore, the study delves into the potential lost diversity in both northern and southern regions. To address the urgent need for effective management and conservation, a deeper understanding of koala population structure and genetic diversity is imperative. By shedding light on the genetic history and current state of koala populations, this research seeks to provide essential genome level insights for effective conservation strategies and ensure the long-term survival of this iconic marsupial species.

## Results

### A Koala Whole-Genome Temporal Dataset

We generated shotgun DNA sequencing data for 93 individual koalas sampled across the historic and contemporary range of the species (except for SA). The historic specimens (*n* = 68) had collection dates between 1817 and 1980. The contemporary 25 samples derived from five extant populations across three Australian states: QLD (n_pop_ = 2), NSW (n_pop_ = 2), and VIC (n_pop_ = 1) (Fig. 1).

We mapped the unprocessed sequencing data to the publicly accessible whole-genome koala assembly v4.1 (GenBank Assembly Accession: GCA_002099425.1, Isolate Bilbo 61053, female, 2017) (Johnson et al. 2018). Individuals with a depth of coverage less than 1× were excluded from subsequent analyses (*n* = 31), resulting in a dataset comprising a total of 37 historic and 25 contemporary whole genomes. For the historic samples, geographic and temporal information was available for 23 and 10 specimens, respectively. Further information on the geographic origins, museum identification numbers and collection dates of all samples are listed in supplementary table S1, Supplementary Material online (also see Fig. 1 for geographic origins of samples).

### Population Structure in Historic and Modern Koalas

**Fig. 2.**
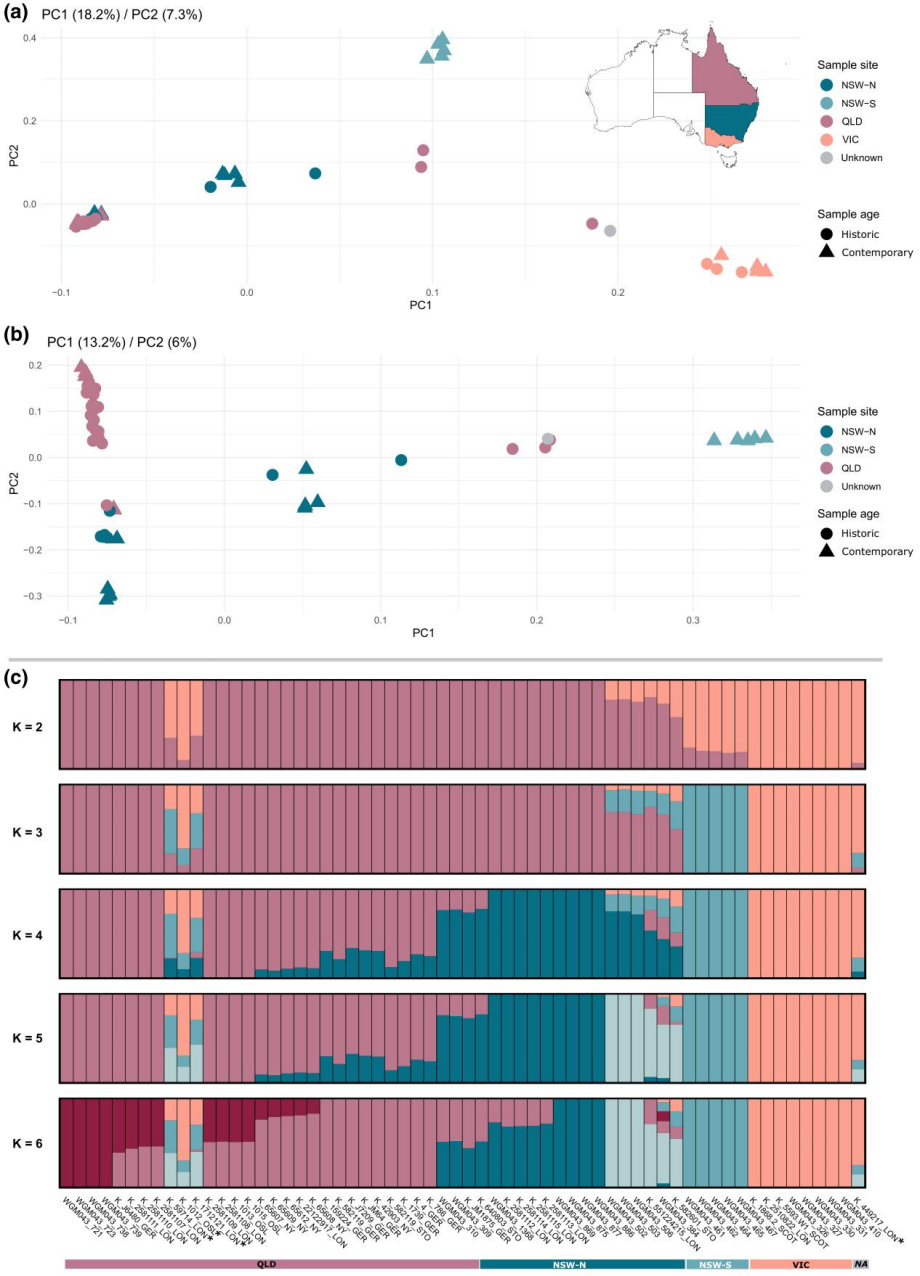
*Population structure of koalas.* PCA of 37 historic and 25 contemporary whole koala genomes from southeastern Australia, including: a) PCA of all 62 koala samples from the three Australian states QLD, NSW, and VIC; b) PCA of 55 koala samples from two southern Australian states (QLD and NSW), and c) Admixture proportion analysis using all 62 koala samples with known and unknown origin. The four historic “outlier” individuals identified by the PCAs are highlighted with asterisks on the admixture plot.

We used the combined historic and contemporary genome dataset, which covers the koala's east and south-eastern range across the Australian continent, to determine the population structure of koalas prior to their decline in numbers in the early 20th century. For the subsequent population structure analyses, we utilized the genotype likelihoods (GLs) derived from variant transversions as our input data. We performed a principal component analysis (PCA) to explore historic and contemporary population structure in koalas (Fig. 2a). The first principal component predominantly arranged samples in a geographic gradient from north to south (QLD, NSW-N, NSW-S, and VIC). However, three historic samples from QLD, along with one historic sample of unknown origin, were positioned unexpectedly among samples from NSW-N, NSW-S, and VIC, deviating from the expected geographic pattern. All contemporary samples formed cohesive clusters alongside their respective historic counterparts. The PCA revealed clear clustering for NSW-S and VIC, while NSW-N and QLD showed signs of admixture or overlap. The second principal component further separated individuals sampled in NSW-S and VIC from those in other localities.

To gain deeper insights into the population structure of koalas within the central and northern regions of their historical and contemporary geographic range, we conducted a second PCA exclusively using QLD, NSW-N, and NSW-S individuals (excluding VIC, as shown in Fig. 2b). In this refined PCA, the north-south gradient of samples, as delineated by PC1, remains distinctly evident. Notably, the peculiarly positioned outlier QLD samples as identified in the initial PCA now form a distinct cluster positioned between the clusters of NSW-S and NSW-N samples. In this second PCA, PC2 serves to further segregate the mixed cluster of QLD and NSW-N samples, leading to the separation of three contemporary individuals from NSW-N. Additionally, a pattern can be observed where most historic and contemporary QLD individuals tend to group together sequentially based on their geographic origin within the state of QLD (supplementary text, Supplementary Material online; supplementary fig. S1, Supplementary Material online).

To investigate the historic and contemporary population structure in more detail, we performed an admixture proportion analysis on the 62 koala genomes and observed a pattern concordant with the results of the PCA analyses (Fig. 2c). A division into two distinct clusters for NSW koalas, following a north-south gradient, became apparent starting with K = 4. The admixture analysis revealed that the historic QLD outlier individuals, detected through the PCA, represent a mosaic of genetic components from NSW-N, NSW-S, and VIC. Three of the historic samples from unknown origins fell into the QLD cluster, whereas the fourth one appeared to closely resemble the three QLD outliers in the genetic structure plot. At K = 2, individuals from VIC in SA were distinguished from those in the north-east (QLD and NSW) (Fig. 2c). For K = 3, NSW-S was distinct from VIC, and at K = 4, NSW-N was separated from other regions. Further, at K = 5, NSW-N divided into two clusters, and at K = 6, individuals from QLD-S were differentiated from QLD-N individuals.

### Mitochondrial Haplotype Network and Phylogenetic Analyses

We bioinformatically obtained the full mitochondrial genome, as well as just the mitochondrial CR from our genomic data, aligned them with all accessible NCBI GenBank mitochondrial and CR sequences for koalas (n = 62_mitochondrion_, n_dloop_ = 53, see supplementary table S2, Supplementary Material online for GenBank Accession numbers), and constructed individual mitochondrial haplotype networks for each of the sequence alignments. This allowed us to place our samples within a broader dataset encompassing the entire geographic range of the species.

**Fig. 3.**
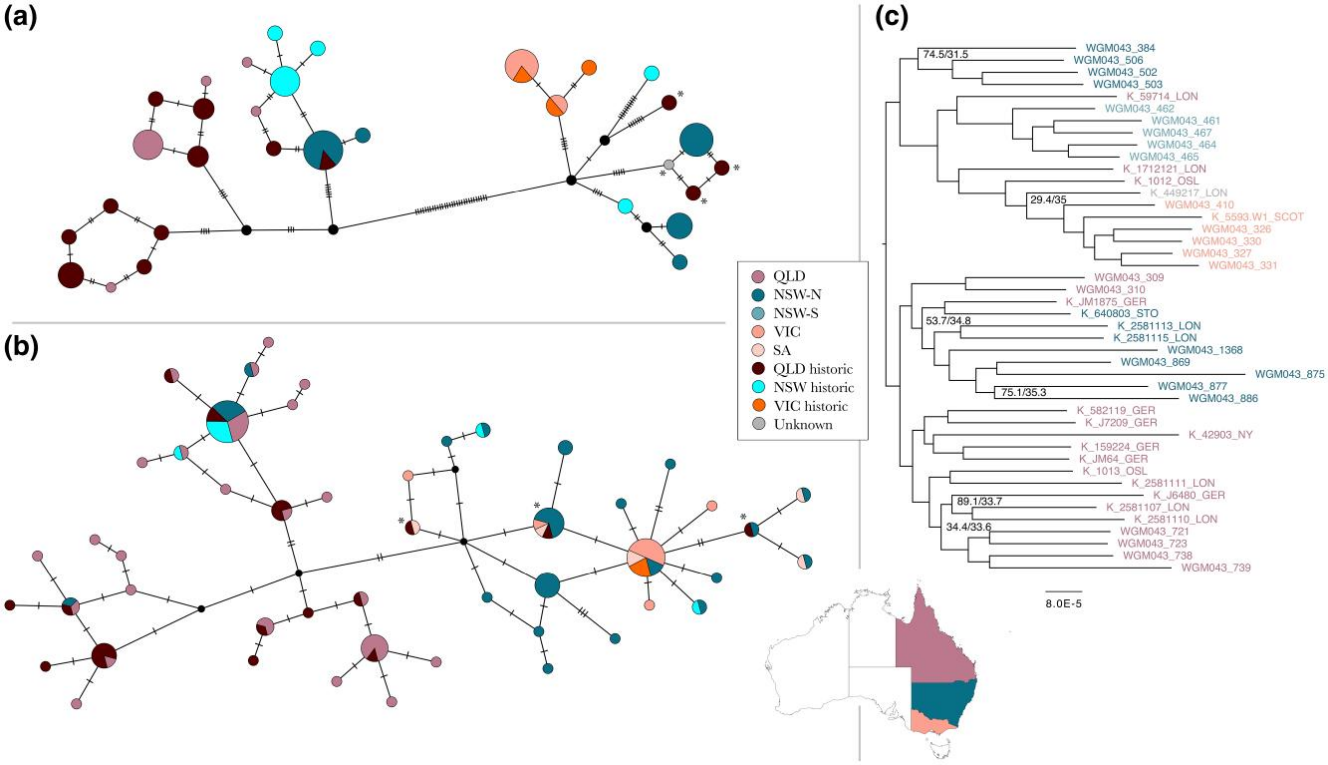
*Mitogenome relationships among koalas.* a) Median Joining haplotype network generated using full mitochondrial genomes retrieved from historic and contemporary koalas investigated in this study (n_mitochondrion_thisstudy_ = 62). b) Median joining haplotype network generated using only mitochondrial CR from the above mentioned mitogenomes (with the exclusion of four individuals due to missing data, n_dloop_thisstudy_ = 58) and including all available NCBI GenBank mitochondrial CR sequence data from other studies (n_dloop_NCBI_ = 53). c) Maximum likelihood genome-scale phylogenetic tree including historic samples of koalas from across their distribution. Branch support is shown for nodes with an aLRT support < 90. Haplotypes that include one or more of the four historic “outlier” individuals identified by the PCAs are highlighted with asterisks.

In the haplotype network based on the whole mitogenomes, all shared haplotypes are shared between individuals from the same geographic region, with the exception of one haplotype that was shared between NSW-N and QLD (Fig. 3a). In this network, three of the four QLD outlier individuals’ group with one haplotype from NSW-S (*K_1712121_LON, K_1012_OSL, K_449217_LON*), whereas the remaining individual clusters with individuals from NSW-N (*K_59714_LON*). The analysis indicates that while historic and contemporary mitochondrial haplotypes from NSW and QLD show slight differences—often differing by only a single mutation—they still cluster closely together within the haplotype network. In contrast, haplotypes from VIC have remained consistent, being the only ones shared between both historic and contemporary samples. The second haplotype network, based solely on the mitochondrial CR, featured three star-like clusters—two encompassing QLD and NSW-N individuals and another comprising exclusively QLD individuals. Additionally, there was a large group of haplotypes representing individuals from all localities. In this network, nearly all historic haplotypes are shared with their contemporary counterparts, except for four unique historic QLD haplotypes, each represented by a single individual. The historic QLD outlier samples identified in the PCA cluster most closely with haplotypes shared by NSW-N, SA, and VIC (Fig. 3b, see asterisks).

We conducted a maximum likelihood phylogenomic analysis on 44 koala samples that had a coverage above 5×, focusing on 22.9 Mb of whole-genome data. This analysis used 1 Mb nonoverlapping windows, filtered to ensure data completeness and to mitigate biases from sequencing errors. The GTR + R model in IQ-TREE2, supported by approximate likelihood ratio tests and site concordance factors, was employed, with quintet rooting applied to the final phylogenetic tree.

In agreement with full mitochondrial haplotype data, nuclear phylogenomic analyses revealed that koalas from VIC are monophyletic, with strong support for the clade (Fig. 3c). Some QLD koalas grouped with northern NSW or Victorian koalas with high support, suggesting admixture among populations. Interestingly, koalas from QLD were not monophyletic, nor were those of northern NSW. There was strong support for this conclusion, suggesting that structuring did not exclusively follow geographic ranges.

We calculated haplotype diversity (Hd) for both historic and contemporary koala individuals using the full mitochondrial sequence alignment, following the formula $Hd=n/n-1(1-\sum p_{i}^{2})$, where *p_i_* is the number of individuals and Hd represents the frequency of each haplotype within the population. The Hd for historic koalas (Hd_historic_ = 0.943) was significantly higher than the Hd for the contemporary koalas (Hd_contemporary_ = 0.876). The resultant *P*-value from the permutation test was 0.017 (supplementary fig. S2, Supplementary Material online).

### Changes in Genomic Diversity Through Time

**Fig. 4.**
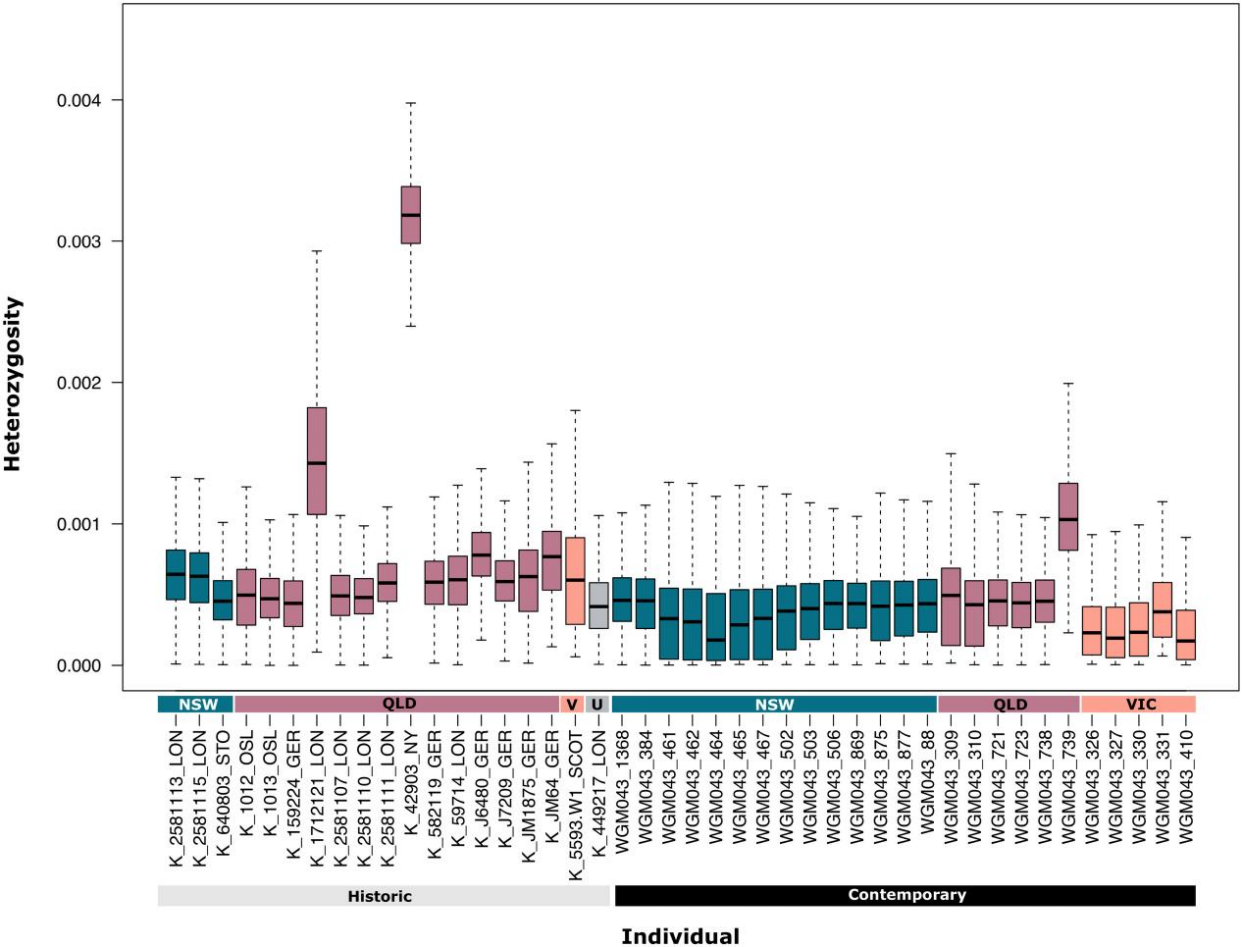
*Genome-wide diversity across time and space.* Individual genomic heterozygosity across three geographically informed koala populations for 25 contemporary and 19 historic samples.

We used two distinct methodologies, namely downsampling and genotype calling with ATLAS, for calculating genome-wide heterozygosity for all 44 koala individuals sequenced to a coverage above 5×. The first method includes coverage correction, which adjusts for differences in sequencing depth by utilizing downsampling, a process that equates data size across samples for comparison (supplementary tables S3 and S4, Supplementary Material online; supplementary fig. S3 to S5, Supplementary Material online). It also adjusts for ancient DNA damage and corrects for sequencing errors (for an overview of DNA damage patterns for our historic samples see supplementary fig. S6, Supplementary Material online). Using a second method, we computed GLs from transversions only, without an error or read depth correction (Fig. 4). In both analyses, across all populations our contemporary individuals exhibit on average lower levels of genome-wide heterozygosity than their historic counterparts. The highest levels of genome-wide heterozygosity were found in individuals from QLD, for both historic and contemporary samples. Contemporary individuals from VIC exhibited the lowest levels of genome-wide heterozygosity. That two different approaches yielded consistent results gives us confidence that there has been a small but noticeable genetic shift over time in these populations. The difference in genome-wide heterozygosity between historic and contemporary koalas was statistically significant across all populations (MeanGwhet_historic_: 0.00144, MeanGwhet_contemporary_: 0.000425, *P*-value: 4.57e-09), for individuals from NSW (*P*-value: 0.013), from QLD (*P*-value = 0.039) but not from VIC (*P*-value = 0.14) using a Kruskal–Wallis nonparametric test in R (supplementary figs. S7 and S8, Supplementary Material online).

**Fig. 5.**
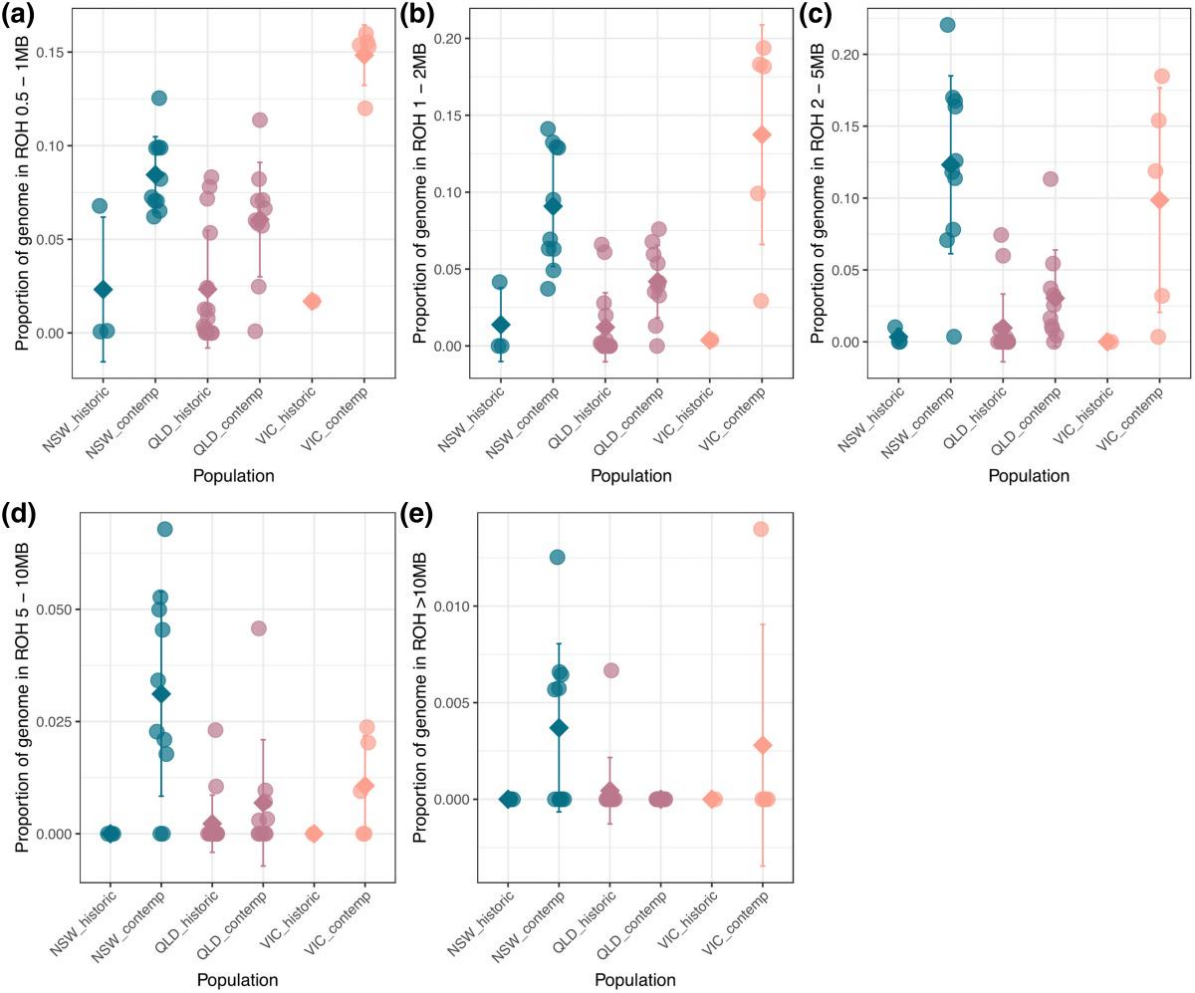
*Inbreeding through time among koalas*. Grouped scatter plots of individual proportions of genome in ROH across three koala populations were divided into size classes to investigate inbreeding at five sequential timeframes of the recent past. Allowing for a generation time of six year equates to inbreeding between 1201 and 2400 year ago (a), 601 to 1200 year ago (b), 241 to 600 year ago (c), 121 to 240 year ago (d), and 0 to 120 year ago (e) for the small, medium, and large F_ROH_ size classes, respectively. NSW, New South Wales; QLD, Queensland; VIC, Victoria. Note: *y*-axis scales can differ between panels (a-e).

We also estimated levels of inbreeding among koalas, by calculating the average length of homozygous regions, known as runs of homozygosity (ROH), and divided it by the total length of scaffolds larger than 10 Mb (see ROH in Methods) to obtain individual inbreeding coefficients (FROH, Fig. 5a-e). Results were plotted into five distinct ROH categories—0.5 to 1 Mb, 1 to 2 Mb, 2 to 5 Mb, 5 to 10 Mb, and over 10 Mb—each indicating a different inbreeding timeframe: ROH at 0.5 Mb suggests inbreeding within the past 200 generations, over 1 Mb within the last 100 generations, over 2 Mb within the past 50 generations, above 5 Mb within the last 20 generations, and exceeding 10 Mb within the last 10 generations. We observed an inverse relationship between the fraction of ROH (FROH) across most window sizes and genome-wide heterozygosity. The lowest FROH values were found in samples from QLD, with NSW following, and the highest in VIC for window sizes ranging from 0.5 to 5 Mb. Intriguingly, contemporary samples from all three states displayed, on average, higher FROH values compared to their historical counterparts. This suggests higher levels of inbreeding in more recent times. However, the disparity is especially marked in shorter window sizes, which is counterintuitive as it suggests an increase in ancient inbreeding events. This pattern could reflect the dynamics of inbreeding and subsequent outbreeding, where initially long ROHs fragment over time through interbreeding among less related individuals, leading to a higher proportion of shorter ROHs in contemporary populations compared to historic ones. The differences between historic and contemporary individuals in their ROHs were highly significant for all window size comparisons under 10 MB (Kruskal–Wallis *P*-value range: 2.29e-06-0.0006) and not significant for windows >10 MB (*P*-value: 0.106) (supplementary fig. S9, Supplementary Material online). Similar to the patterns we noted in genome-wide heterozygosity, the observed increasing FROH further highlight a potential shift in inbreeding trends over time within these populations.

### Intraspecific Long-term and Short-term Demographic Histories

**Fig. 6.**
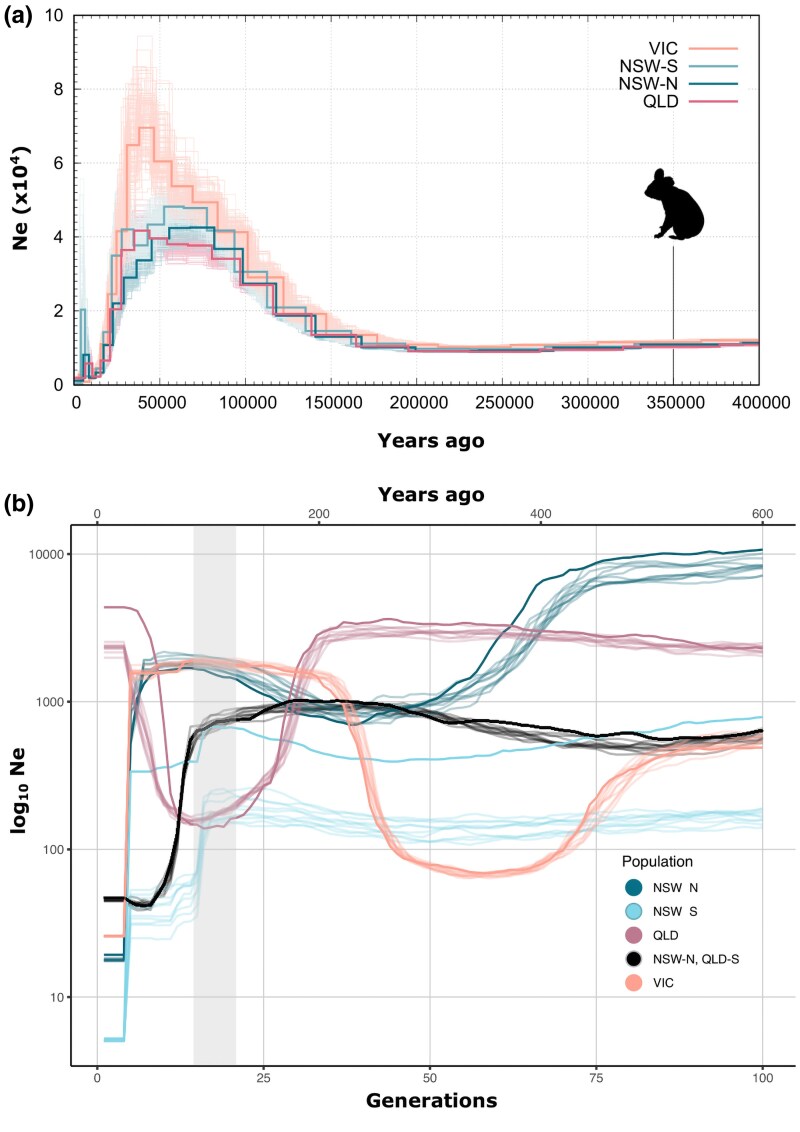
*Demographic history of koalas.* a) Inference of effective population size using the PSMC method for four geographically informed koala populations. The representation of the earliest fossil record of modern koala (Anon 2014) is symbolized by the koala silhouette. Three (WGM043_327_VIC: 12.3x, WGM043_465_NSW-S: 10.9x, WGM043_502_NSW-N: 11.4) out of the four represented populations were downsampled to the lowest coverage of the fourth contemporary individual (WGM043_721_QLD-N: 9.9x). b) GONE for the same koala populations as in (a) with the addition of admixed individuals from QLD-S and NSW-N, plotted for a generation time of six years (Santiago et al. 2020). Bolt lines show the results of the five GONE analyses that ran for each population on the maximum number of SNPs (50,000), while faded lines represent the ten independent GONE runs, with each subsampling 40,000 SNPs. The culling period is highlighted in grey.

For a long-term demographic perspective, going back hundreds of thousands of years, we conducted a pairwise sequential Markovian coalescent (PSMC) analysis on 4 koala genomes from all Australian states represented in this study (QLD (*n* = 1), NSW (*n* = 2), and VIC (*n* = 1)). Three of the four populations (WGM043_327_VIC: 12.3x, WGM043_465_NSW-S: 10.9x, WGM043_502_NSW-N: 11.4x) were downsampled to match the lowest coverage level of the 4th contemporary individual (WGM043_721_QLD-N: 9.9x) to correct for potential biases caused by coverage deviations. The investigation of the koala population's demographic history reveals that the earliest lineage to diverge was the Southern lineage (VIC), estimated to have occurred ∼230,000 to 250,000 year ago (Fig. 6a). Subsequent lineage splits occurred around 200,000 year ago. Notably, the trend line trajectories for the other three lineages—NSW-N, NSW-S, and QLD—are nearly overlapping and show minimal differences from one another. A notable population decline is observed across all lineages, commencing between 30,000 and 50,000 year ago, with the earliest decline observed in NSW-N and the latest in NSW-S and QLD-N/S.

To elucidate the more recent demographic history of the same koala populations, including admixed individuals from the border region between the states of QLD and NSW (NSW-N, QLD-S), we ran a genetic optimization for N_e_ estimation (GONE) analysis (Fig. 6b). The GONE results show that individuals from VIC experienced a distinct bottleneck ∼300 to 500 year ago (ya), with a sharp increase in population numbers around 300 to 200 ya. The only other population with a similar, yet less severe trajectory was NSW-N. This population showed a considerable decline around 400 ya and did not fully recover to its previous effective population size. The QLD population remained stable until ∼200 ya, and then experienced a significant drop, which seems to be on an upward trajectory for the last ∼80 year. During the intensive hunting period beginning of the 20th century (Fig. 6b, gray bar) only the populations NSW-S and QLD-S; NSW-N show a small but distinct drop in N_e_. The N_e_ of both populations has since been on a steady downward trend according to our GONE analysis.

## Discussion

Our study marks the first comparison of whole genomes of both historic and contemporary koalas from across the eastern range of the species, revealing critical insights into the genetic impacts of historical and contemporary human activities, notably hunting for the fur trade. When analyzing the 37 historic and 25 contemporary genomes, we observed a pronounced decrease in genome-wide heterozygosity and a loss of both mitochondrial and nuclear diversity in modern koala populations compared to their historical counterparts. This loss in genetic diversity might indicate a reduction in present day koalas, potentially compromising the population's resilience to environmental changes and diseases.

### Historic and Contemporary Population Structure of the Koala

The principal component, as well as the admixture analysis of koalas from the three Australian states of QLD, NSW, and VIC revealed a clear geographic structuring of koala populations along a north-south axis, with distinct clustering corresponding to the geographic origins of the koalas (QLD, NSW-N, NSW-S, and VIC). The interspersion of historic QLD samples within the NSW and VIC clusters hints at historical gene flow among these populations, a connectivity that is less evident in contemporary samples. The Plio-Pleistocene biogeographic barriers that are believed to underlie contemporary genetic structure in koalas are primarily coastal (Neaves et al. 2016; Johnson et al. 2018; Lott et al. 2024). It is therefore possible that, prior to the urbanization of native ecosystems and the associated fragmentation and ongoing eastward contraction of suitable koala habitat, some western edge populations may have enjoyed greater connectivity across ancestral biogeographic barriers than conspecifics on the coast. Alternatively, undocumented human mediated translocations may have allowed certain genotypes to expand into areas where they would otherwise not be expected to occur. The history of koala translocations across eastern Australia is not well recorded, and there is evidence for translocations by organizations such as the Acclimatisation Society of Victoria in the late 1800s and, more recently, by local governments, wildlife carers, and other organizations (Fowler et al. 2000; Wedrowicz et al. 2017). This understandably complicates the identification of historic and contemporary biogeographic barriers and dispersal corridors. Nevertheless, the geographic patterning of genetic diversity identified by our analyses, and the formation of cohesive clusters among contemporary samples alongside their historic counterparts, underscore the enduring impact of regional isolation and possible effects of human activities on koala genetics over time. Further exploration via a second PCA (Fig. 2b), limited to koalas from central and northern regions, not only recapitulated the north-south gradient but also brought to light a distinct QLD cluster. This pattern has become more pronounced, as in the previous PCA analysis, the presence of southern individuals skewed and stretched the samples, masking this cluster's visibility, which is now clearly observable with the exclusion of Southern individuals. This supports the existence of a distinct genetic lineage in QLD, which, while evident in the historic dataset, appears absent in the contemporary genetic landscape. The admixture analysis complements these findings by showing that historic QLD outlier individuals possess a genetic makeup indicative of a blend from NSW and VIC populations. This could suggest historic intermixing or a shared genetic heritage that has since been obscured by population declines and habitat fragmentation or it could be indicative of the inability of the analysis to find a unique genetic cluster for these individuals due to low sample size and the use of the minimal minor allele frequency (MAF) threshold. The division of NSW koalas into two clusters and the distinct genetic partition of QLD koalas, as revealed by the admixture analysis, reflect a complex population structure potentially influenced by both natural biogeographic barriers and more recent anthropogenic impacts. The presence of historic QLD outliers with mixed genetic components underscores the potential for genetic diversity that may have been eroded over time.

Our mitochondrial haplotype networks and phylogenomic analysis corroborated these findings. Mitochondrial haplotype networks highlighted the geographic maternal structuring of populations, with notable genetic links between NSW-N and QLD koalas. This structuring was further confirmed through phylogenomic analysis, which revealed distinct clades within QLD and VIC, alongside evidence of past interbreeding between QLD, northern NSW, and Victorian populations, as well as recent gene flow between southern QLD and northern NSW koala populations. It is noteworthy that the topology of the phylogeny aligns closely with the major genetic clusters as identified in the studies by Johnson et al. (2018) and Lott et al. (2022) with the latter study also incorporating historical samples. Our analyses underscore a complex genetic tapestry, suggesting historically higher gene flow and genetic diversity across koala populations.

### Historic and Contemporary Genomic Diversity of the Koala

Our study further revealed substantial changes in koala genomic diversity over time through two methodologies calculating genome-wide heterozygosity. The first method, incorporating coverage correction and adjusting for ancient DNA damage and sequencing errors, alongside a second method focusing on transversions without error correction, consistently showed contemporary koalas displaying lower heterozygosity compared to historic populations. Notably, individuals from QLD maintained the highest heterozygosity levels, while koalas sourced from VIC had the lowest. While lower genetic diversity is typically associated with increased disease susceptibility, southern koala populations exhibit lower genetic diversity alongside lower disease prevalence, which seems counterintuitive. One possible explanation is that, despite reduced genetic diversity, certain advantageous alleles—perhaps related to immune function—have become fixed in these populations, offering protection against diseases like Chlamydia and the KoRV (Robbins et al. 2020). This could result from historical population bottlenecks selecting for individuals with stronger immunity. Supporting this idea, southern koalas exhibit very low levels of full-length KoRV, reducing the health threat posed by this retrovirus, as well as a lower prevalence of Chlamydia compared to northern populations (Speight et al. 2013). In contrast, northern populations, with higher genetic diversity, may retain more genetic variation, including alleles that increase susceptibility to these pathogens. While this is outside the scope of the present study, future research exploring specific immune gene variants in these populations could shed light on this intriguing pattern.

Inbreeding levels, assessed through ROH and resulting in individual inbreeding coefficients (FROH), displayed an inverse relationship with genome-wide heterozygosity. This relationship confirms the genetic consequences of inbreeding, as regions of homozygosity increase when genetic diversity decreases (Kardos et al. 2018a, 2018b). Contemporary samples generally exhibited higher FROH values, suggesting a recent increase in inbreeding events, marking a potential shift in inbreeding patterns within these populations over time. It is important to note that substantial shifts in genomic diversity often take multiple generations to manifest, even after severe bottleneck events (Browne et al. 2019). Given the generation time of koalas (∼6 to 8 year; Ellis et al. 2010), the full genomic consequences of historical overhunting or habitat fragmentation may not yet be fully apparent. This aligns with the timeline of koala population declines, which were particularly severe during the late 19th and early 20th centuries due to overhunting and habitat destruction (Lunney et al. 2012). The time elapsed since these events suggest that the long-term genetic impacts are only beginning to emerge. ROHs >10 Mb reflect inbreeding events occurring roughly within the past 60 to 80 year, corresponding to ∼10 generations. This timeframe overlaps with the period following the population declines caused by human activity and is consistent with population fragmentation and size reductions, which limit genetic diversity and increase the likelihood of mating between close relatives (Frankham et al. 2010). However, several confounding factors may influence our interpretation of the ROH data. For instance, regional differences in population size, connectivity, and environmental pressures could contribute to the observed patterns (Hoffmann and Sgrò 2011). Additionally, the sensitivity of ROH detection methods may limit our ability to capture the full extent of inbreeding over shorter or more fragmented genomic regions (Ceballos et al. 2018). This limitation arises because shorter ROHs, which are indicative of more ancient inbreeding events, may be more difficult to detect due to their subtle genomic signatures and their potential fragmentation over time (Kardos et al. 2018a, 2018b). Low marker density or sequencing depth may fail to resolve smaller ROHs, as their size approaches the lower limit of what most ROH detection tools can reliably identify, especially if there are gaps or heterozygous interruptions within what was once a contiguous ROH, resulting in underestimation of ancient inbreeding (Howrigan et al. 2011; Ceballos et al. 2018). These factors collectively highlight the challenges in accurately assessing the complete historical inbreeding patterns within populations.

### Evolutionary History of the Koala

Long-term demographic history reconstructed through PSMC analysis identified the Southern lineage (VIC) as the first to diverge around 230,000 to 250,000 year ago. The other genetically distinct groups of koalas appear to have emerged relatively quickly after this initial divergence, with all extant lineages being detectable by ∼150 to 200 kya. This timeframe is broadly concordant with the findings of previous studies using reduced genomic datasets (Lott et al. 2022), and provides further evidence that the emergence of contemporary koala population structure has been driven by vicariance associated with increased glacial-interglacial cyclicity in the Middle Pleistocene. In contrast to previous studies, our PSMC results did not detect evidence of multiple distinct lineages, apart from VIC (Kjeldsen et al. 2016; Lott et al. 2022), in the long-term demographic history trends. However, when examining more recent demographic history using GONE, clear differences were observed between all lineages. This could indicate that the lineages in QLD and NSW diverged too recently (<20 kya) for PSMC to detect (Westbury et al. 2023), or that they experienced similar demographic trajectories due to shared ancestry and/or comparable environmental pressures (Fraebel et al. 2017). However, all lineages showed major population declines starting between 30,000 and 50,000 year ago. While the causes of these population declines are likely to be complex and multifactorial, it is highly probable that the arrival of humans in Australia (48 to 50 kya; Allen and O'Connell 2020) played at least a contributory role. Recent genomic studies have demonstrated a near ubiquitous decline in global megafauna populations between 32 and 76 kya (Bergman et al. 2023). Furthermore, both genomic and macroecological analyses have consistently identified a significant role of human biogeography in global patterns of megafauna extinction, with climate exerting a relatively minor or even negligible influence (Svenning et al. 2024). Although koalas are generally not large enough to be classified as megafauna, the concurrent decline of all major genetic lineages within the same approximate timespan suggests that this species was also negatively affected by human expansion. While active hunting may have led to a reduction in koala numbers in certain areas, the near simultaneous, continent-wide declines uncovered by our analyses suggests that large-scale factors, such as vegetation reorganization caused by anthropogenic fire regimes or the selective loss of browse-dependent megafauna, exerted the greatest negative impact on the species (Miller et al. 2005). It is highly probable that refugia on the east-coast of Australia played a significant role in the survival of koalas through the continent-wide loss of suitable habitat that is believed to have occurred during the late Pleistocene (Adams-Hosking et al. 2011; Black et al. 2014). The species is known to have once been much more geographically widespread, with fossil deposits containing koalas appearing in all Australian states except Tasmania and the Northern Territory (Black et al. 2014). The last record of koalas in Western Australia (WA) comes from Devil's Lair and has been dated to around 31 to 43 kya, a timeline roughly concordant with the arrival of humans in the area (Balme et al. 1978). However, it should also be noted that, at least toward the western edge of the koala's distribution, the replacement of eucalypt forests with a mosaic of open habitats, including semiarid woodland and shrubland, likely began much earlier (>100 kya; Black et al 2014). The eastward contraction of core koala habitat is hypothesized to have reached its peak during the last glacial maximum (GLM), with independent bioclimatic modeling suggesting that the species was restricted to geographically limited areas of northern NSW and QLD, and small areas of coastal Western Australia, South Australia, and VIC (Adams-Hosking et al 2011). Consequently, the high levels of genomic diversity observed in QLD koalas relative to their conspecifics further south may indicate that the greater amount of suitable habitat that persisted in this state may have supported larger, better connected koala populations, which endured comparatively minor demographic bottleneck events. It cannot be overlooked, however, that the PSMC clearly demonstrated that extant koala lineages had already undergone significant declines in effective population size well before the last GLM. This, coupled with the apparent ability of koalas to weather previous climatic fluctuations (i.e. the pronounced glacial–interglacial cycles of the middle Pleistocene) with no detectable negative demographic consequences, strongly implies that climate shifts of the late Pleistocene alone cannot explain the continent-wide declines in koala population numbers that occurred prior to European-colonization of Australia.

An investigation of more recent demographic history using GONE revealed considerable variation in temporal patterns of effective population size across the koala's range, based on analyses of modern, un-admixed individuals. Populations in both northern NSW and VIC appear to have experienced major bottlenecks beginning ∼400 to 500 year ago. While both groups show evidence of subsequent recovery, the NSW koala populations in particular never returned to pre-bottleneck numbers. Similarly, koalas in QLD underwent notable population declines beginning ∼200 year ago. While the primary causes of these demographic shifts are currently unclear, it has been hypothesized that koalas have experienced cyclical population expansions and contractions throughout the Quaternary, depending on factors such as the climate, the availability of preferred tree species, and, following the arrival of humans on the Australian continent, the intensity of hunting (Tsangaras et al. 2012; McAlpine et al. 2015). As these pressures are not temporally or geographically uniform across the koala's distribution, it follows that, at various times, different groups or lineages would have been disproportionately impacted. Interestingly, only koalas in southern NSW showed evidence of significant declines in effective population size during the period of intense hunting that took place in the late 19th and early 20th centuries. While the other lineages have also experienced substantial population bottlenecks over the past 110 year (with the exception of QLD, which appears to be trending upward) these effects were often not evident until decades after hunting had been outlawed. It is not unusual for substantial shifts in genomic diversity to take several generations to occur, even after severe bottleneck events (Browne et al. 2019). Given the generation time of koalas (6 to 8 years), it is perhaps unsurprising that the genomic consequences of overhunting would not be immediately apparent. By contrast, the historically low effective population size of the southern NSW lineage, possibly reflecting a more scattered and disjunct distribution, may have exacerbated the impacts of overhunting and accelerated the appearance of negative genomic consequences. Alternatively, the precipitous decline observed in the effective population sizes of most koala lineages over the past century may be linked to causes other than overhunting (e.g. climate change or habitat loss). This suggests that maintaining large census population sizes might be less crucial for the genomic health of wild koalas than ensuring the availability of suitable habitats that support effective dispersal. Hence preserving, and restoring koala habitats may be more vital for their long-term genetic health than focusing solely on increasing population numbers. Corridors of native habitat facilitating gene flow may also explain the increasing effective population size of the QLD lineage, despite the substantial and well documented declines in census population sizes across much of the state (de Villiers 2015; McAlpine et al. 2015). It should be noted that neither of the explanations presented above are mutually exclusive, and that both overhunting and anthropogenic environmental modification may have played significant roles in the reduction of contemporary koala populations.

### Management Implications

Climate change and anthropogenic habitat loss are expected to further reduce connectivity between koala populations, leading to an increasingly fragmented and disjunct distribution (Black et al. 2014; Adams-Hosking et al. 2016). As natural dispersal routes are lost or irrevocably altered, active management interventions will become even more necessary to facilitate gene flow and maintain the evolutionary viability of the species. Population monitoring is widely recognized as a critical component of such threatened species management paradigms (Frankham 2015; Ralls et al. 2018). Information collected through DNA-based monitoring programs can be used to better understand spatial and temporal population dynamics and evaluate both local and species-level responses to specific management actions (de Barba et al. 2010). Long-term monitoring is of particular importance following translocations or reintroductions, in order to ensure that such actions achieve their stated goal of maintaining or increasing genomic diversity (Robinson et al. 2020). Our results demonstrate that all extant koala lineages have endured repeated population declines as a consequence of climatic and anthropogenic influences. While this suggests that koalas are likely to continue to be negatively impacted by ongoing environmental disturbances in the Holocene, it also provides some hope that affected populations will be able to persist and even recover, provided that adequate steps are taken to mitigate the elevated risks of extinction associated with inbreeding and the loss of genomic diversity. Widespread evidence of past admixture between vicariant koala lineages suggests that genetic rescue (i.e. the translocation of individuals between genetically divergent populations) is a viable conservation strategy for this species. While more research is required to predict the net genomic effects of translocations between specific populations, the movement of individuals between groups that represent the same lineages should be encouraged where possible, provided that appropriate precautions are also taken to reduce the likelihood of negative nongenetic effects (e.g. disease transmission; Woodford and Rossiter 1993; Dalziel et al. 2017). Although koala translocations should be seen as a last resort, they are becoming more frequent, e.g. in NSW, due to challenges in habitat protection and compromised natural dispersal routes (New South Wales Government 2022).

However, our findings also demonstrate at least one potential pitfall of employing DNA-based monitoring to inform management actions. The GONE analysis suggests that there can be substantial delays between major demographic or stochastic events and detectable genomic consequences. This means that even rigorous monitoring efforts may fail to identify koala populations that require targeted interventions before the effects of key threatening processes become difficult or impossible, to counteract. In extreme cases, this could even contribute to extirpation if critical infrastructure and resources are directed away from seemingly stable koala populations due to the genomic consequences of severe demographic declines evading immediate detection. One explanation could be that when severely bottlenecked populations do bounce back, rapid increases in population size are in fact mitigating genomic erosion (Jackson et al. 2022). This process enhances genetic diversity and reduces inbreeding effects, making the adverse impacts of the bottleneck harder to detect. Consequently, meaningful conservation outcomes will only be achieved if active management interventions are complemented by the development of more robust legislation and management frameworks which address the root causes of continuing koala population declines, particularly drought and the loss of critical native habitats.

In conclusion, our study not only sheds light on the historical and contemporary genetic landscape of koalas but also raises urgent calls for integrating genetic considerations into conservation planning. By understanding the genetic and demographic histories of koala populations, we can tailor conservation efforts to mitigate the impacts of past actions and ensure the future viability of this iconic species amidst ongoing environmental and anthropogenic challenges.

## Materials and Methods

### Sampling, DNA Extraction, Library Preparation, and Sequencing for Historical Samples

The historical samples analyzed included material obtained from 68 museum specimens from museums in Australia, Germany, England, United States of America, Scotland, Sweden, and Norway. Collection dates ranged between 1817 and 1980. Samples consisted of keratinous material (pieces of skin and hairs), or bone powder. Samples were stored and processed in facilities dedicated to ancient DNA work at the Globe Institute (Copenhagen) and the Natural History Museum in London (NHMUK).

Between 10 to 100 mg of each sample was processed, following two different DNA extraction protocols. Samples from specimens from the Mammal Collection at the NHMUK were extracted following a version of the extraction protocol described in Brace et al. (2019). Briefly, in the digestion stage, 360 μL of Qiagen ATL Buffer and 20 μL Proteinase K were added to each sample and incubated with rotation at 56 °C for 24 h. DNA purification followed the protocol by Dabney et al. (2013) but replacing the Zymo-Spin V column binding apparatus with the extender assembly from the High Pure Viral Nucleic Acid Large Volume Kit (Roche). All the other samples from the collections of the Australian Museum (AM), QLD Museum, Kansas University Museum, Cambridge Museum of Comparative Zoology, Royal Ontario Museum, National Museums Scotland (NMS), Swedish Museum of Natural History (SMNH), University of Oslo Museum of Natural History (NHMUK), and the American Museum of Natural History (AMNH) were processed following the protocol described in Barnett et al. (2018). Briefly, samples were digested overnight (ca. 16 h) using a Proteinase K containing buffer (Gilbert et al. 2007). The digest was centrifuged, and the supernatant was collected and mixed with 8 × of a binding buffer as detailed in Allentoft et al. (2015) then centrifuged through Monarch DNA Cleanup Columns (5 μg) (New England Biolabs Inc. Beverly, MA, USA). DNA bound to the columns was washed with 800 μL buffer PE (Qiagen, Hilden, Germany), then eluted in 25 μL buffer EB (Qiagen).

The DNA extracts resulting from both extraction approaches were subsequently built into genomic libraries using a modified version of the BEST-single tube protocol (Carøe et al. 2018), optimized for highly degraded and chemically complex DNA extracts. Briefly, DNA extract was end-repaired in a 50 μL reaction consisting of 7.5 U T4 DNA polymerase, 10 U T4 PNK dNTP, 1 × T4 DNA ligase buffer (NEB), 0.25 mM dNTPs and 2.75 μL of “Reaction enhancer” (25% poly-ethylene glycol (PEG4000); 2 μg/μL Bovine Serum Albumin (BSA) and 400 mM NaCl) and incubated for 30 min at 20 °C, followed by purification using Monarch DNA Cleanup Columns (5 μg), using 750 μL buffer PB (Qiagen), washed with 800 μL buffer PE (Qiagen), and eluted in 33.8 μL of buffer EB (Qiagen). The resulting DNA was adapter-ligated in a 50 μL reaction consisting of 400 U T4 DNA ligase, 1 × T4 DNA ligase buffer (NEB), 6.25% PEG-400, 2.2 µL of “Reaction enhancer” and BGI-SEQ specific adapters as described by Mak et al. (2018). The reaction was incubated for 30 min at 20 °C and 10 min at 65 °C and purified using Monarch DNA Cleanup Columns as above. Finally, the fill-in reaction was carried out using Bst 2.0 Warmstart polymerase. The reaction was incubated for 15 min at 65 °C and 15 min at 80 °C and purified using Monarch DNA Cleanup Columns as above.

Quantitative real-time PCR (qPCR) was used to estimate the required number of cycles for library index PCR. Each qPCR was performed in a 20 μL reaction volume using 1 μL of purified library template, 1 × KAPA HotStart ReadyMix, 1 μL SYBR Green (Invitrogen, Carlsbad, CA, USA), 0.3 μM forward and reverse primers and 1 μL of BSA (NEB). qPCR cycling conditions were 98 °C for 2 min, followed by 40 cycles of 98 °C for 30 s, 60 °C for 60 s, and 72 °C for 60 s using the MX3005 qPCR machine (Agilent). Post-qPCR, library index amplifications were performed in 50 μL PCR reactions using 15 μL of purified library template, 1 × KAPA HotStart ReadyMix, 0.3 μM forward and reverse index primers and 1 μL of BSA (NEB). Cycling conditions were as above but with the number of cycles previously estimated for each sample. PCR reactions were purified with 1.4 × of AmpureXP beads and incubated at RT for 5 min, followed by 2 washes of fresh 80% EtOH, before drying the reaction for 3 min and eluting in 32 μL of EB buffer with 5 min incubation at 37 °C.

The libraries for all 68 specimens were shotgun sequenced at BGI-Europe to assess level of preservation and estimate clonality and endogenous content. Shotgun statistics revealed endogenous DNA ranging from 0% to 90%. Based on this, individuals with > 5% endogenous content (*n* = 37) were further sequenced to a final depth of coverage ranging from 1.5× to 16.5×.

### Sampling, DNA Extraction, Library Preparation, and Sequencing for Modern Samples

We obtained biological material (ear clips) from the AM Koala Tissue Biobank, from 25 modern koala specimens, representing individuals from each of the five major genetic clusters identified by Johnson et al. (2018) and Lott et al. (2022) (supplementary table S1, Supplementary Material online). We extracted genomic DNA using either the Bioline Isolate II Genomic DNA Kit (Bioline, Eveleigh, Australia) following the manufacturer's protocols, or a standard high-salt precipitation procedure (Sunnucks and Hales 1996). We assessed integrity and concentration of the DNA using a Genomic Screentape on the Agilent 4200 Tapestation (Agilent Technologies, Mulgrave, Australia) according to the manufacturer's instructions. Genome library construction and sequencing was performed using the BGISEQ-500 platform using BGI Australia's commercial service (∼30 Gb of data per sample).

### Bioinformatic Data Processing and Quality Assessment

#### Quality Check and Mapping of DNA Sequencing Data

We performed a raw data quality check per sample with fastqc v0.11.7 (Andrews 2010). We then used the pipeline PALEOMIX 1.2.13.2 (Schubert et al. 2014) to map the raw sequencing reads of the historic specimens against the whole-genome *P. cinereus* assembly v4.1(GenBank Assembly Accession: GCA_002099425.1, Isolate Bilbo 61053, female, 2017) (Johnson et al. 2018). Within PALEOMIX, we trimmed adapter sequences, stretches of Ns, and low-quality bases and filtered them with AdapterRemovalv2 (Lindgreen 2012; Schubert et al. 2016) using default parameters. We used BWA v0.7.17 (Li and Durbin 2009) mem to map the cleaned reads to the koala genome, with default parameters. We filtered reads with mapping quality of <30 using SAMtools v1.6 (Li et al. 2009). We removed duplicates with picard v2.6.0 (Broad institute 2019). After this, we filtered out possible paralogs using SAMtools. Finally, we performed local realignment around indels using GATK v3.3 (McKenna et al. 2010) and base quality scores were adjusted around aDNA damage patterns using mapDamagev2 (Jónsson et al. 2013).

For the modern specimens, we trimmed adapter sequences and removed short (<30 bp) reads using skewer v0.2.2 (Jiang et al. 2014). We merged overlapping read pairs using FLASH v1.2.11 (Magoč and Salzberg 2011; Danecek et al. 2021). Both merged and unmerged reads were mapped to the koala reference genomes using BWA, the mem algorithm and otherwise default parameters. We removed PCR duplicates and reads with mapping qualities <30 using SAMtools.

#### Generation of Consensus Genome Sequence per Sample

A consensus sequence file was generated in*.fasta* format for each of the*.bam* files retrieved from mapping using ANGSD v 0.921(Korneliussen et al. 2014). For the data mapped to the mitochondrial reference, the consensus base per site was chosen, (option -doFasta 2), while for whole-genome mapped data, a random base per site was drawn (option -doFasta 1). The following quality filtering parameters were used when building the consensus sequences: -minQ 30 -minMapQ 30 -uniqueonly 1 -docounts1.

#### Sex Scaffold Identification

We identified putative sex chromosomes using satsuma synteny (Grabherr et al. 2010) by aligning the koala reference genome against the human X (NC_000023.11) and Y (NC_000024.10) chromosomes. All aligning scaffolds were removed from further analyses.

### Population Structure Analyses

#### Principal Component Analysis

We generated a PCA on all 62 historic and contemporary koalas, using GLs computed with ANGSD v0.921 (Korneliussen et al. 2014), specifying only scaffolds >100 kb (-rf), -minq 30, -minmapq 30, -domajorminor 1, -dohaplocall 2, -docounts 1, -uniqueonly 1, -gl 2, -minMaf 0.05, -minind 31, -doGlf 3, -SNP_pval 1e-6, -rmtrans 1, and -doMaf 1. We converted the resultant GL into a covariance matrix using PCAngsd v0.98 (Meisner and Albrechtsen 2018). We then removed all individuals originating from VIC (*n* = 7) and ran a second PCA using the same parameters, except for -minind 25. We plotted the covariance matrices in R (https://www.R-project.org/) with ggplot and plotly (Sievert 2020).

### Admixture Analysis

#### NGSadmix

We evaluated the mixed ancestry proportions in individuals using NGSadmix v32 (Skotte et al. 2013), utilizing transversion variant sites from the GLs of 62 koala genomes computed earlier for the PCA. To explore ancestral clusters (K) within a range from two to six, we performed 100 iterations of NGSadmix for each K value. We selected the highest log-likelihood run for each K for visualization with the software Pong (Behr et al. 2016).

### Mitochondrial Haplotype Network and Phylogenetic Analyses

We bioinformatically obtained the complete mitochondrial genome from our genomic data when mapping to the koala reference genome, specifying chromosome “MT” as the mitochondrial reference (NCBI Reference Sequence: NC_012682.1). Initially, all 93 samples, including those excluded from other analyses due to low autosomal coverage (<1×), were screened for valuable mitogenome data. However, out of the 31 additional low-coverage samples assessed, only five met the quality threshold for inclusion. Unfortunately, these five museum samples lacked precise metadata, such as their geographic origin, and as a result, we opted not to include them in the analysis. The mitochondrial CR from our genomic data (*n* = 62_dloop_thisstudy_) was then extracted by aligning it to a consensus sequence of the CR, obtained from a multiple sequence alignment of all available sequences downloaded from NCBI. We aligned our mitochondrial and *dloop* sequences with all available NCBI GenBank mitochondrial and CR sequences for koalas (*n* = 62_mitochondrion_thisstudy_, *n* = 62_dloop_thisstudy_, *n* = 53_dloop_NCBI_; see supplementary table S2, Supplementary Material online) using BWA v0.7.17 aln for the 38 historic samples and BWA v0.7.17 mem for the 25 contemporary koala samples. We then constructed individual mitochondrial Median Joining haplotype networks for each of the sequence alignments (mitochondrion, dloop). We visualized the quality of the mitochondrial genomes using Geneious Prime 2022.1.1 (https://www.geneious.com) after importing the BAM files. We then constructed individual mitochondrial Median Joining haplotype networks for each alignment using PopART (Leigh and Bryant 2015).

We performed phylogenomic analysis on whole-genome data, excluding historic samples with coverage below 5x. We extracted nonoverlapping 1 Mb windows from the whole-genome alignment, resulting in 2,925 regions. From each, we then extracted the first 10 Kb for further phylogenomic analysis. We filtered each window to remove any samples with data in <20% of sites and excluded alignment sites where <80% of the samples contained data. This filtering approach, based on data completeness, helps to mitigate biases from sequencing errors and uncertain data alignment segments. Additionally, we excluded all sites containing only transitions from the alignments to avoid frequent DNA damage in historic samples. Our final phylogenomic dataset included 44 koala samples and covered 22.9 Mb. We conducted a maximum likelihood phylogenetic analysis on this concatenated alignment using the GTR + R model in IQ-TREE2 (Minh et al. 2020b), with four categories of free rates (Kalyaanamoorthy et al. 2017). For branch support estimates, we used approximate likelihood ratio tests (Guindon et al. 2010) and site concordance factors (Guindon et al. 2010; Minh et al. 2020a). Finally, we applied quintet rooting to the tree, as it outperforms other common fast rooting approaches (Tabatabaee et al. 2022).

### Genomic Diversity Analyses

#### Genome-wide Heterozygosity

ATLAS and downsampling (theta): We selected a historic (K_640803) and a contemporary (WGM043_327) koala genome with the highest coverages (16.8 × and 12.3 × respectively) to be downsampled to investigate the role of coverage in heterozygosity estimates. We downsampled the historic individual to 12.3 × to make it comparable to the modern individual. We downsampled to five different coverages (25%, 40%, 50%, 60%, 80% of the original 12.3×) and repeated this independently three times (supplementary table S3, Supplementary Material online). Downsampling was performed using SAMtools. We calculated an error correction for each of the resulting downsampled datasets (15 per individual), with ATLAS (task = recal) utilizing the haploid mitochondrial genome with a minimum base quality threshold of 30 (minQual = 30) to determine sequencing error rates. The mitochondrial genome was specifically chosen because “recal” relies on haploid sites to train and develop recalibration parameters, which are then applicable for genome-wide error correction. We also calculated an aDNA error correction in ATLAS on the historic individual. Heterozygosity was calculated for each downsampled dataset using ATLAS (task = estimateTheta) with the previously established recalibration parameters, maintaining the base quality threshold (30), and limiting the analysis to scaffolds >100 kb in length. The relative heterozygosity compared to the “authentic” heterozygosity discerned at the various downsampling levels was graphed, under the assumption that 12.3 × coverage accurately reflects authentic heterozygosity. A third-order polynomial regression was applied (supplementary fig. S3, Supplementary Material online; supplementary table S4, Supplementary Material online) to derive a formula to account for heterozygosity biases due to reduced coverage in contemporary samples. The resulting correction formula is (0.0008×coverage^3) − (0.0265×coverage^2) + (0.2998×coverage) + 0.2191. To correct for false positive heterozygosity in the historic individuals, the resultant polynomial regression line with the equation (0.0063×coverage^2) − (0.1399×coverage) + 1.781 was applied (supplementary fig. S4, Supplementary Material online; supplementary table S4, Supplementary Material online). It is important to note that these correction formulas are likely specific to the particular software, parameters, and data used, and should be applied to other datasets and programs with careful consideration.

We also computed site allele frequencies using GLs on the scaffolds > 100 kb for each individual >5 × coverage using GLs in ANGSD and the following parameters: domajorminor 1, GL 2, noTrans 1, mininddepth 5, minmapq 30, minq 30, uniqueonly 1, doSaf 1, fold 1. We then converted site allele frequencies into heterozygosity values using the realSFS tool in the ANGSD tool suite in 1 Mb windows (-nsites) and a tolerance of 1e-08.

### Runs of Homozygosity

We restricted our analysis for the ROH estimation to scaffolds >10 Mbp in the koala reference assembly, after removing the previously identified sex scaffolds. This resulted in 102 scaffolds in total. We generated a PLINK file using ANGSD v0.935 (-doPlink 2), including only individuals with coverage above 5×, following the approach used by Foote et al. (2021). We specified the parameters: -minq 30, -minmapq 30, -domajorminor 1, -doGeno −4, -doPost 1, -gl 2, -postCutoff 0.95, -docounts 1, -uniqueonly 1, -minMaf 0.05, -nthreads 10, -minind 22, -SNP_pval 1e-6, -rmtrans 1, and -doMaf 1. We then processed the resulting PLINK file using PLINK to determine ROH, applying parameters such as –homozyg-snp 50, –homozyg-kb 1000, –homozyg-gap 1000, and –allow-extra-chr. To compute individual inbreeding coefficients (FROH), we divided the cumulative length of ROH segments exceeding 1 Mbp by the total base pairs in scaffolds longer than 10 Mb.

We separated the output into five distinct ROH classes as follows: 0.5 Mb - 1 Mb, 1 Mb - 2 Mb, 2 Mb - 5 Mb, 5 Mb - 10 Mb, and > 10 Mb. To estimate the number of generations since inbreeding occurred, the formula g = 100/(2rL) was used, as proposed by Kardos et al. (2018a, 2018b), where “r” represents the recombination rate, “L” is the length of ROH in megabases (Mb), and “g” signifies the number of generations. Based on this calculation and assuming a recombination rate of 1 cM/Mb (Johnson et al. 2018), ROH at 0.5 Mb suggests inbreeding within the past 200 generations, ROH >1 Mb points to inbreeding within the last 100 generations, ROH over 2 Mb indicates inbreeding within the past 50 generations, ROH above 5 Mb implies inbreeding within the last 20 generations, and ROH exceeding 10 Mb suggests inbreeding occurred within the last 10 generations.

### Demographic History Analyses

#### Pairwise Sequentially Markovian Coalescent Method (PSMC)

We conducted a demographic analysis on five diploid consensus genomes representative of the identified genomic population clusters in this study using PSMC (Li and Durbin 2011). We generated diploid genome sequences via SAMtools and BCFtools, setting a minimum quality score of 30. Before initiating PSMC, we excluded scaffolds aligned with sex chromosomes and those shorter than 100 kb. We ran PSMC with the parameters -N25 -t15 -r5 -*P* “4 + 25*2 + 4 + 6” and performed 100 bootstrap replicates to investigate support for the resultant demography. To ensure the PSMC results were not overfit, we verified that a minimum of ten recombination events were inferred across the intervals after 20 rounds of iteration. We visualized the data using a mutation rate of 1.905 × 10−8 per site per generation, based on a koala generation time of six years (Santiago et al. 2020).

### High-resolution Analysis of Linkage Disequilibrium, GONE

We applied the GONE method (Santiago et al. 2020) to determine the recent effective population size (Ne). This approach involves assessing linkage disequilibrium (LD) across various SNP pairs, taking into account different recombination rates, to identify the sequence of Ne values that most accurately reflects the observed LD pattern (Santiago et al. 2020). We categorized our contemporary koala samples into five populations based on geographic origins and applied GONE to each population separately. We excluded admixed individuals identified during the Admixture analysis from this analysis. We created ped- and map-files using PLINK v.1.9.0, excluding scaffolds aligned with sex chromosomes and those shorter than 10 Mbp for each of the population subsets. When running GONE, we used the default parameters, which included no MAF pruning, exclusion of SNPs that were not genotyped, a maximum recombination rate of 0.05, and 40 iterations for internal replication. We did not employ phasing. Due to the unknown recombination rate for the species, we used the recommended rate of 1 cM/Mb (Santiago et al. 2020). This estimation could potentially be on the higher side, considering that marsupials typically exhibit a lower recombination rate compared to other vertebrates (Deakin 2018). When we executed GONE over 200 generations, the Ne estimates were unrealistically high, but they became substantially lower and more realistic when set to run over 2,000 generations (default option). Consequently, we spanned our runs over 2,000 generations but restricted reporting to findings from the latest 200 generations, as this period is most accurate for detecting Ne fluctuations (Santiago et al. 2020). We calculated Ne for the 2,000-generation span in increments of every five generations, using 400 bins. To evaluate the variability of Ne estimates, we conducted ten separate runs, each with a new subset of 50,000 SNPs, akin to bootstrapping.

## Supplementary Material

msaf057_Supplementary_Data

## Data Availability

The sequencing data underlying this article is available on SRA under BioProject Number PRJNA1198700.
