# Supplementary material for: Road to Extinction? Past and Present Population Structure and Genomic Diversity in the Koala
Source: Mol Biol Evol. 2025 Mar 25;42(4):msaf057. doi: 10.1093/molbev/msaf057 (PMC12014528; doi:10.1093/molbev/msaf057)
Supplement: msaf057_Supplementary_Data [file msaf057_supplementary_data.zip › Supplementary Material_final.pdf]

Supplementary material of *Road to extinction? Past and present population structure and genomic diversity in the koala*

Binia De Cahsan, Marcela Sandoval Velasco, Michael V Westbury, David A Duchêne, Mikkel H Strander Sinding, Hernán E Morales, Daniela C Kalthoff, Ian Barnes, Selina Brace, Roberto Portela Miguez, Alfred L Roca, Alex D Greenwood, Rebecca N Johnson, Matthew J Lott, M Thomas P Gilbert

Table S1. Overview of the number and origin of koala samples in the dataset. *NA* - Not available/unknown

Table S2. NCBI Accession numbers of all 53 downloaded mitochondrial control region (d-loop) sequences of *Phascolarctos cinereus* used to create a multiple sequence alignment.

Table S3. Downsampling and calculation of theta coverage corrections for contemporary and historic koala samples: We selected a historic koala genome (K_640803) and a contemporary koala genome (WGM043_327) with the highest coverage levels (16.8x and 12.3x, respectively) for downsampling to examine the impact of coverage on heterozygosity estimates. The historic sample was downsampled to 12.3x to match the coverage of the contemporary sample. We performed downsampling to five different coverage levels (25%, 40%, 50%, 60%, and 80% of the original 12.3x) and repeated this process independently three times. The relative heterozygosity, compared to the "authentic" heterozygosity observed at various downsampling levels, was plotted, assuming that 12.3x coverage accurately reflects true heterozygosity. A third-order polynomial regression was used to derive a formula to correct for heterozygosity biases resulting from reduced coverage in contemporary samples. The resulting correction formula is (0.0008 x coverage^3) - (0.0265 x coverage^2) + (0.2998 x coverage) + 0.2191. To correct for false positive heterozygosity in the historic individuals, the resultant polynomial regression line with the equation (0.0063 x coverage^2) - (0.1399 x coverage) + 1.781 was applied.

Table S4. Theta coverage corrections for contemporary and historic koala samples using the calculated correction factor from STable 3.

Table S5. Mean genome-wide heterozygosity for 25 contemporary and 19 historic koala samples computed with angsd and R

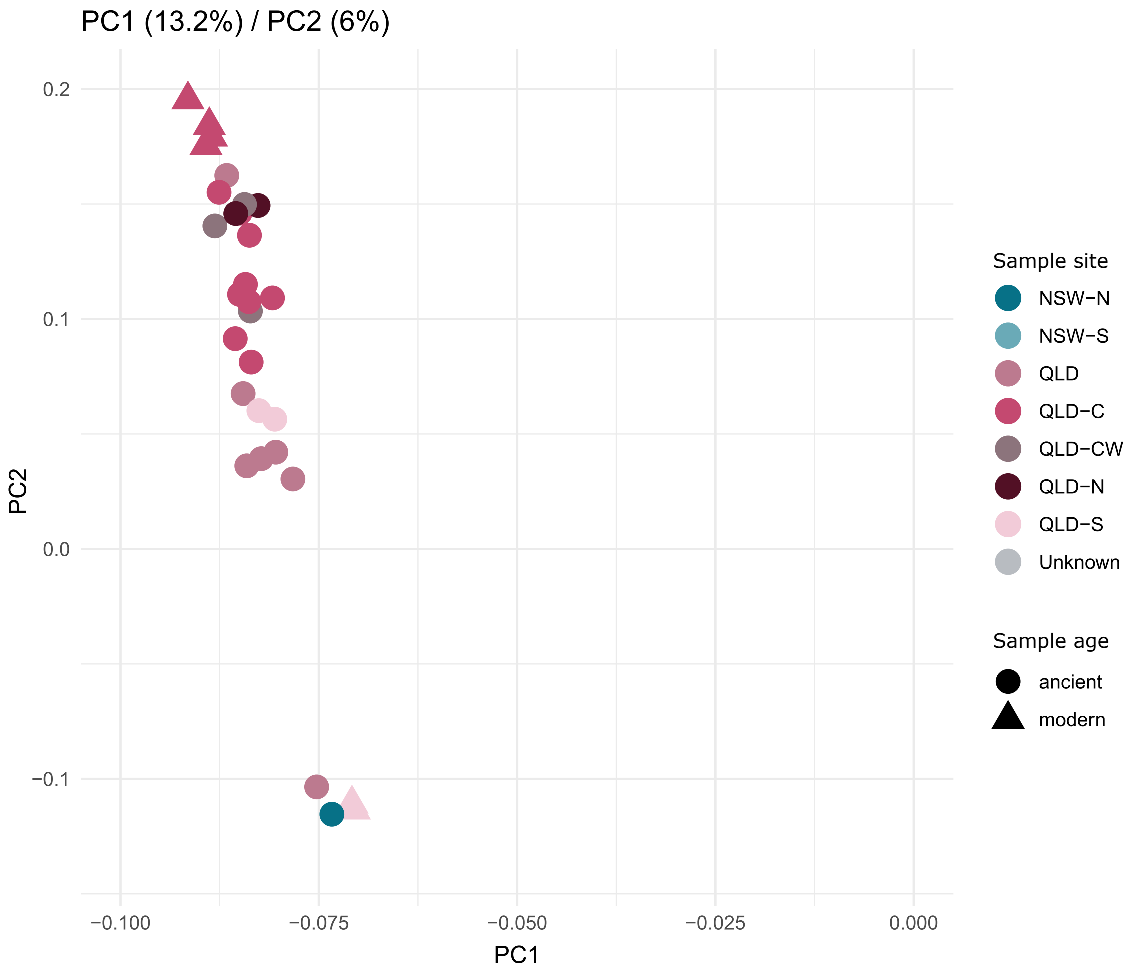

Fig. S1: *Population structure of koalas.* Zoom in on Principal Component Analysis (PCA) of 29 historic and contemporary whole koala genomes from northeastern Australia (Mainly QLD). NSW-N: New South Wales North, NSW-S: New South Wales South, QLD: Queensland (no GPS available), QLD-C: Queensland Central, QLD-CW: Queensland Central West, QLD-N: Queensland North, QLD-S: Queensland South.

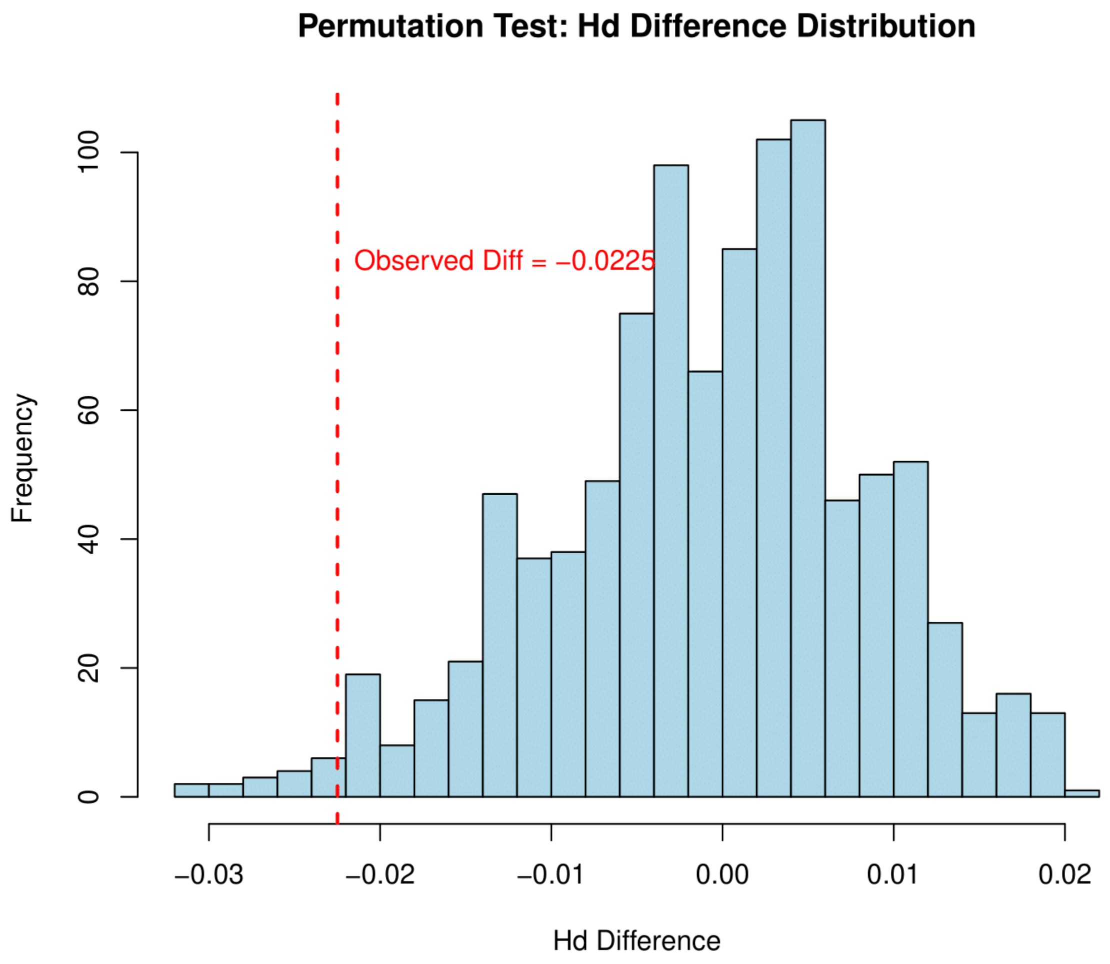

Fig. S2: Permutation test distribution of haplotype diversity (Hd) differences between contemporary and historic mitochondrial koala sequences. The histogram shows the distribution of differences in haplotype diversity (Hd) values between contemporary and historic koala individuals generated from 1,000 permutations in R. In each permutation, group labels were randomly shuffled, and the Hd values were recalculated to simulate the null hypothesis of no difference between the groups. The red dashed line represents the observed Hd difference between the contemporary and historic koala individuals based on the original data. The position of the observed difference outside the distribution indicates the magnitude of the difference relative to the permuted values. The calculated p-value for the permutation test is 0.01698302.

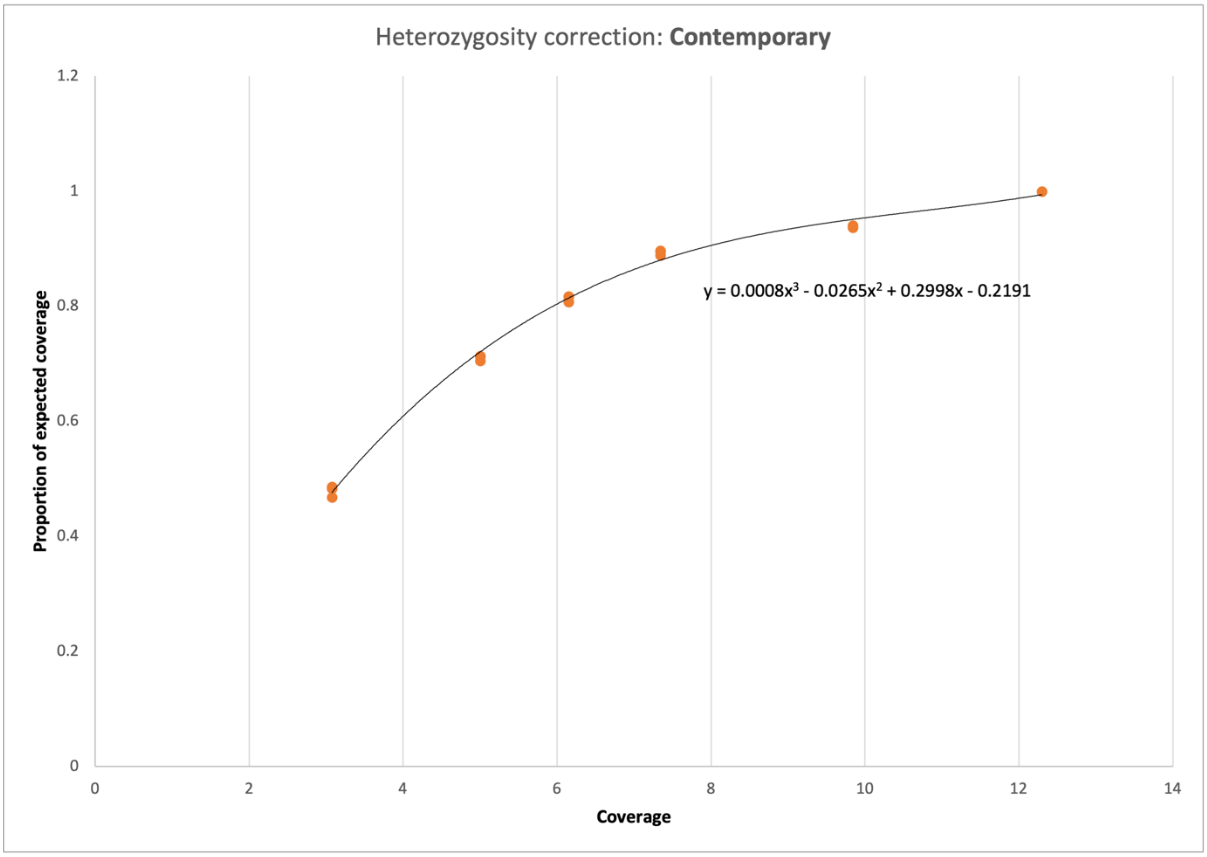

Fig. S3: A third-order polynomial regression was applied to derive a formula to account for heterozygosity biases due to reduced coverage in contemporary koala samples. Graph of relative heterozygosity compared to "authentic" heterozygosity discerned at various downsampling levels, assuming that 12.3x coverage accurately reflects authentic heterozygosity.

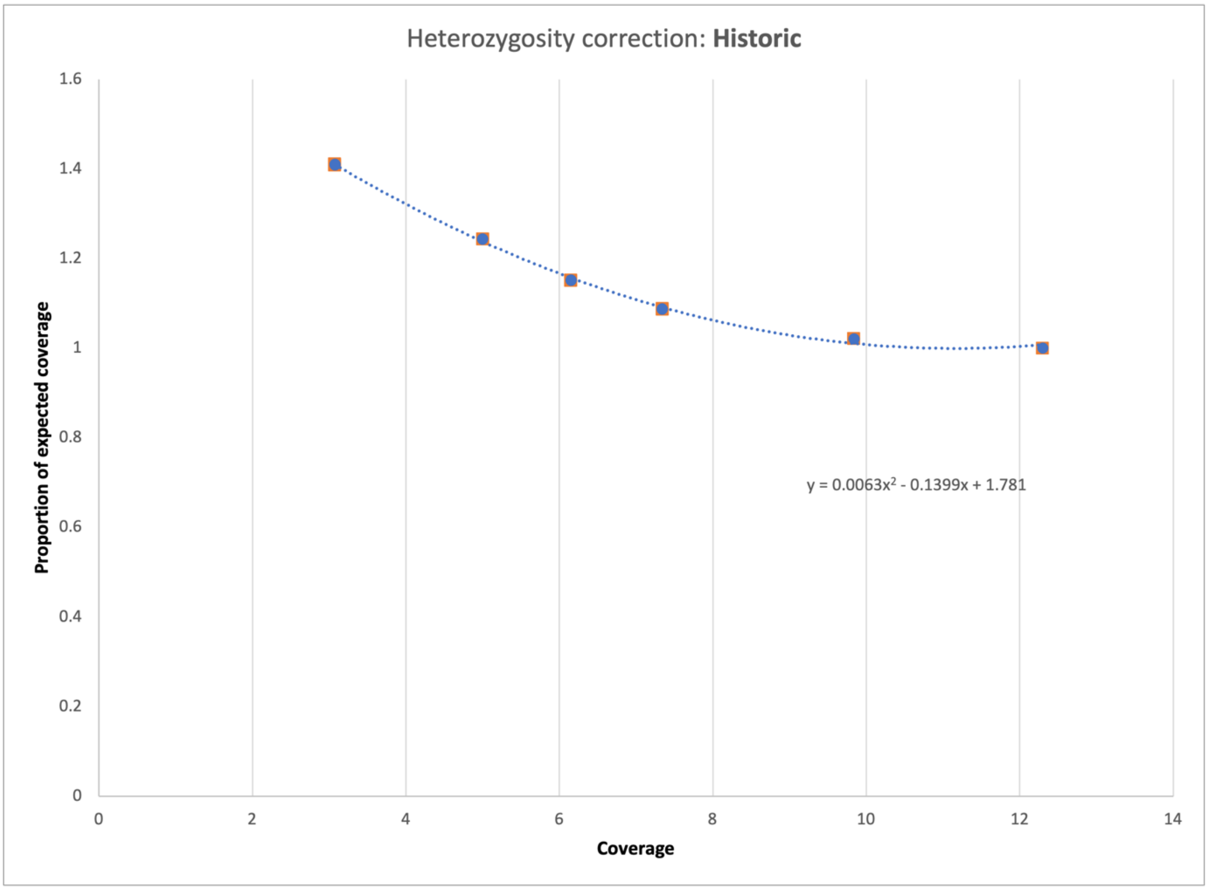

Fig. S4: A third-order polynomial regression was applied to derive a formula to account for heterozygosity biases due to reduced coverage in historic koala samples. Graph of relative heterozygosity compared to "authentic" heterozygosity discerned at various downsampling levels, assuming that 12.3x coverage accurately reflects authentic heterozygosity.

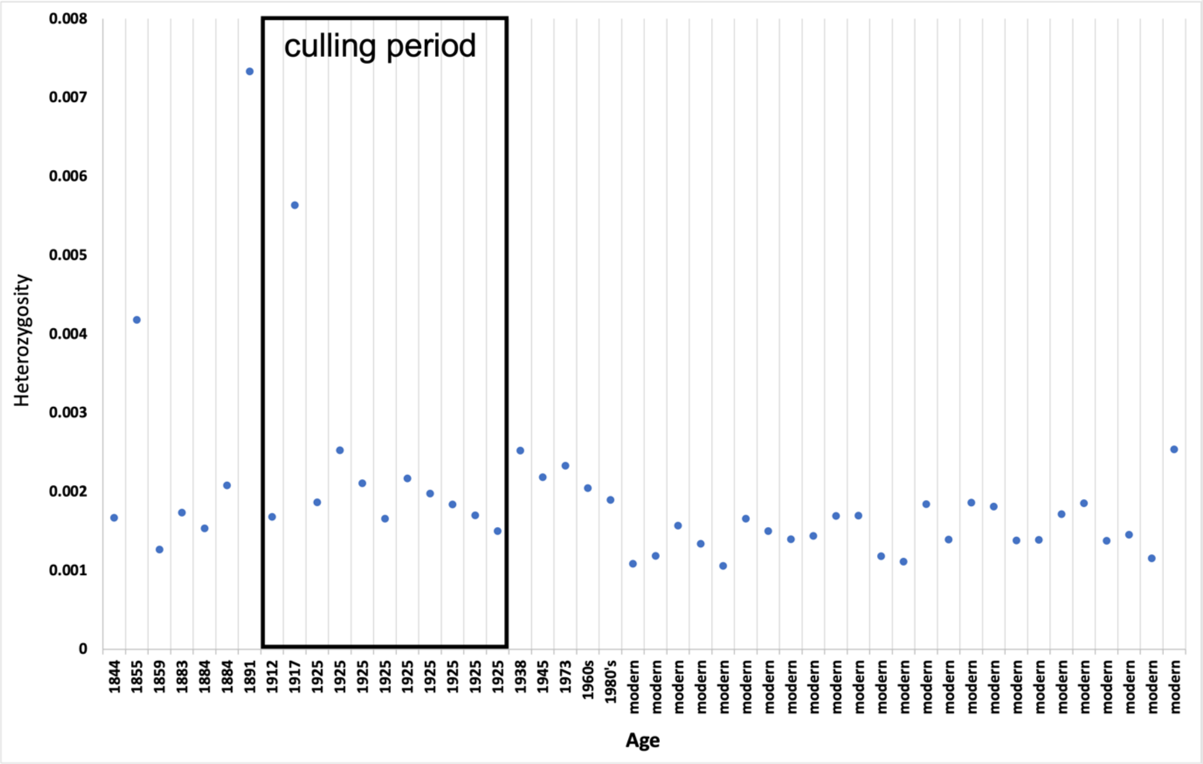

Fig. S5: *Genome-wide diversity across time and space***.** Individual genomic heterozygosity across three geographically informed koala populations after coverage correction calculated with ATLAS.

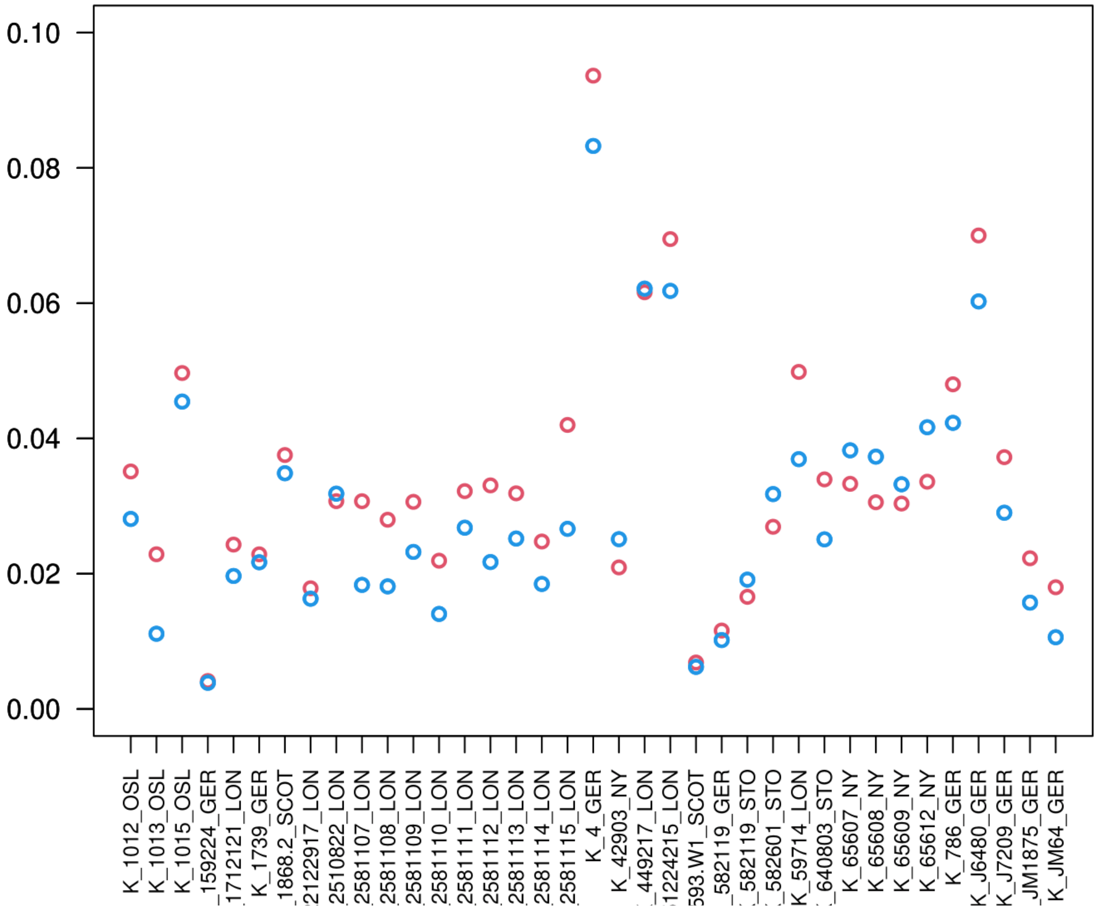

Fig. S6: DNA damage results taken from Mapdamage for all historic koala individuals. The frequency of C-T transitions on the first site from the 5-prime end of the read is shown in red, and G-A transitions on the first site from the 3-prime end of the read is shown in blue.

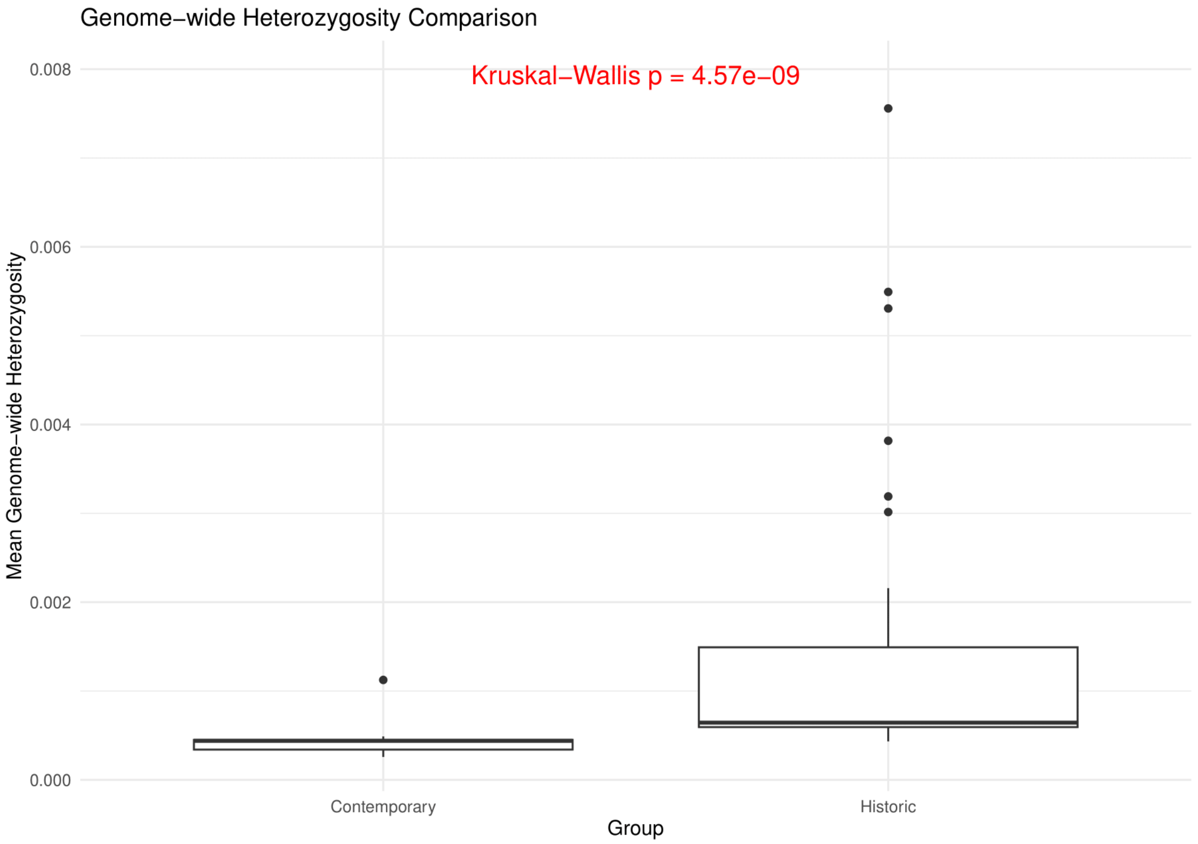

Fig. S7: Significance test for genome-wide heterozygosity comparison between historic and contemporary koala individuals from three different populations. The non-parametric Kruskal-Wallis test was performed using R (v4.2.1) to assess differences in heterozygosity between the two time periods. The p-value from the Kruskal-Wallis test is highlighted in red.

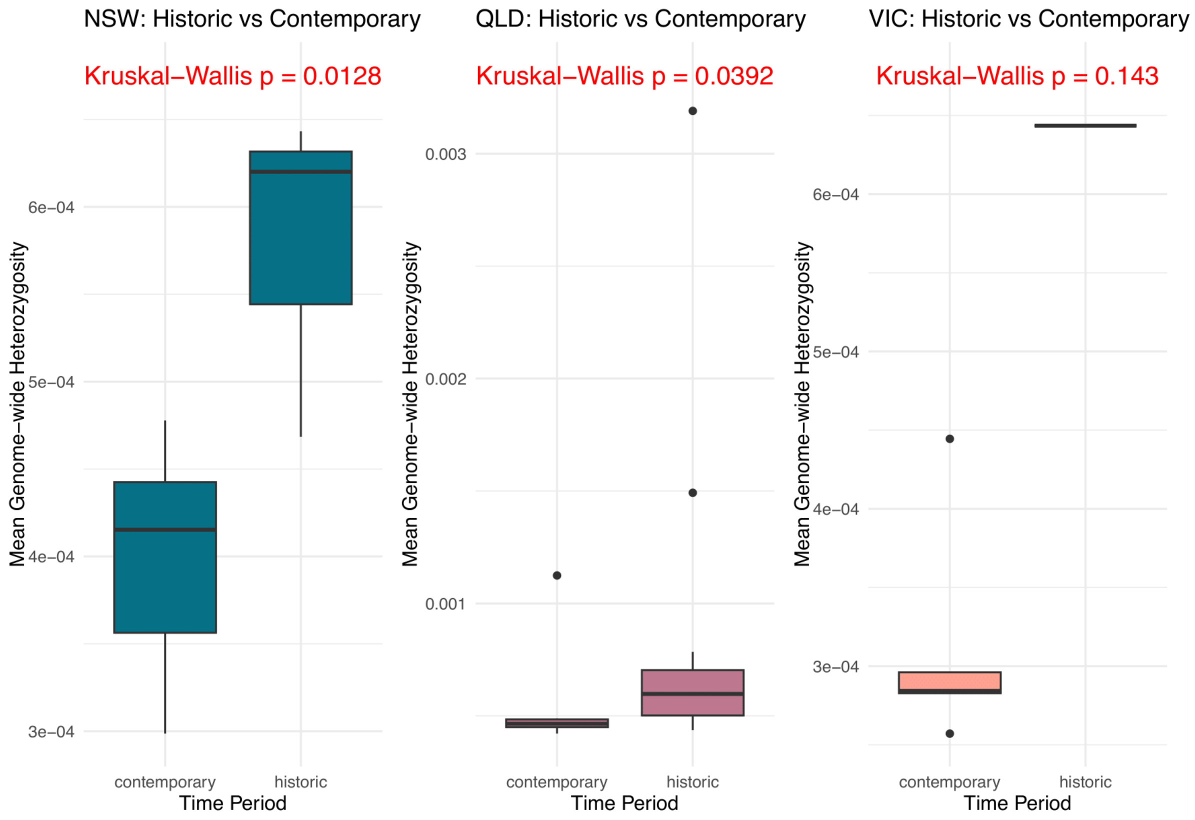

Fig. S8: Genome-wide heterozygosity comparison between historic and contemporary populations of koalas from three regions: New South Wales (NSW), Queensland (QLD), and Victoria (VIC). Each boxplot represents the distribution of mean genome-wide heterozygosity for individuals from historic and modern time periods. The non-parametric Kruskal-Wallis test was performed using R (v4.2.1) to assess differences in heterozygosity between the time periods for each region. The p-values from the Kruskal-Wallis tests are highlighted in red on each plot for the respective comparisons.

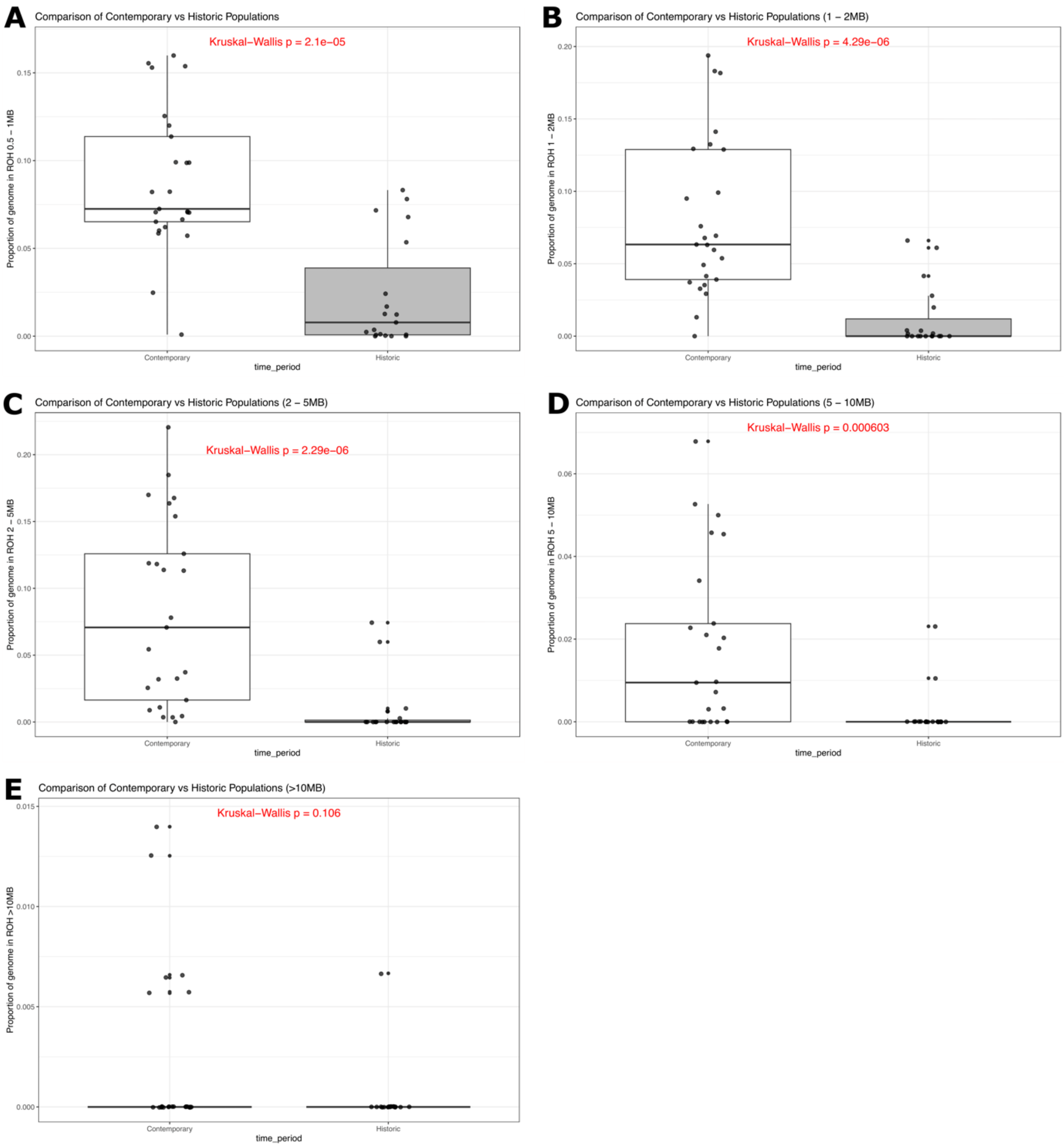

Fig. S9: Comparison of genome-wide Runs of Homozygosity (ROH) across historic and contemporary populations of koalas from Queensland (QLD), New South Wales (NSW), and Victoria (VIC). The five panels show the proportion of the genome in ROH across different size categories: (A) ROH between 0.5 - 1 MB, (B) ROH between 1 - 2 MB, (C) ROH between 2 - 5 MB, (D) ROH between 5 - 10 MB, and (E) ROH larger than 10 MB. For each panel, individual points represent the proportion of genome in ROH for each population, with historic populations compared to their modern counterparts. The Kruskal-Wallis p-values displayed in each panel indicate the statistical significance of the difference in ROH proportions between the two time periods.

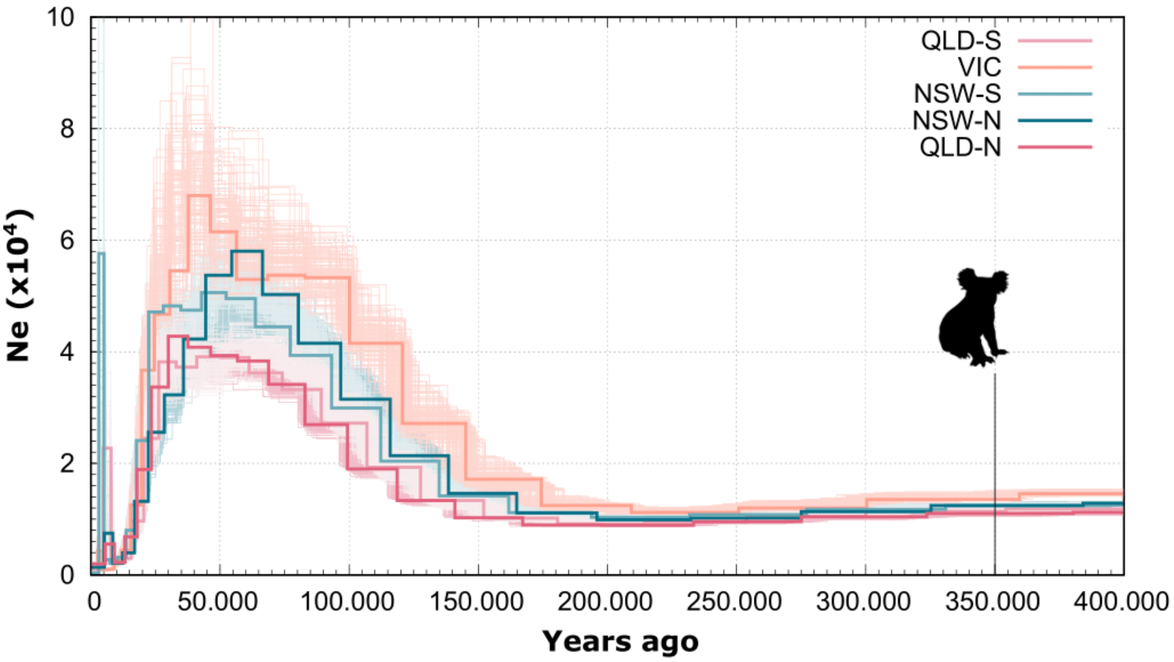

Fig. S10: *Demographic history of koalas.* Inference of effective population size using the pairwise sequential Markovian coalescent (PSMC) method for five geographically informed koala populations (QLD-N, QLD-S, NSW-N, NSW-S, VIC) without correcting for differences in low coverage using a downsampling approach. The representation of the earliest fossil record of modern koala (Anon 2014) is symbolised by the koala silhouette.
